# A CRISPR/Cas9-based central processing unit to program complex logic computation in human cells

**DOI:** 10.1073/pnas.1821740116

**Published:** 2019-03-28

**Authors:** Hyojin Kim, Daniel Bojar, Martin Fussenegger

**Affiliations:** ^a^Department of Biosystems Science and Engineering, ETH Zürich, CH-4058 Basel, Switzerland;; ^b^Faculty of Science, University of Basel, CH-4058 Basel, Switzerland

**Keywords:** synthetic biology, biocomputing, genetic engineering

## Abstract

Controlling gene expression with sophisticated logic gates has been and remains one of the central aims of synthetic biology. However, conventional implementations of biocomputers use central processing units (CPUs) assembled from multiple protein-based gene switches, limiting the programming flexibility and complexity that can be achieved within single cells. Here, we introduce a CRISPR/Cas9-based core processor that enables different sets of user-defined guide RNA inputs to program a single transcriptional regulator (dCas9-KRAB) to perform a wide range of bitwise computations, from simple Boolean logic gates to arithmetic operations such as the half adder. Furthermore, we built a dual-core CPU combining two orthogonal core processors in a single cell. In principle, human cells integrating multiple orthogonal CRISPR/Cas9-based core processors could offer enormous computational capacity.

## Significance

By enabling rational programming of mammalian cell behavior, synthetic biology is driving innovation across biomedical applications. Using Cas9-variants as core as protein-based central processing units (CPUs) that control gene expression in response to single-guide RNAs as genetic software, we have programmed scalable Boolean logic computations such as the half adder in single human cells. Combining orthogonal Cas9-variants enabled the design of multicore genetic CPUs that provide parallel arithmetic computations. The Cas9-based multicore CPU design may provide opportunities in single-cell mammalian biocomputing to provide biomedical applications.

In the physiological context, cells sense environmental inputs, such as metabolites, growth factors, or bacterial toxins, and respond through outputs, such as development, differentiation, or an immune response. These outputs are modulated via the regulation of specific gene switches by intrinsic programmed gene circuits. On the other hand, from the viewpoint of synthetic biology, the idea of introducing synthetic gene circuits into cells to achieve a range of desired nonphysiological outputs opens up many exciting possibilities, including the use of suitably engineered cells to conduct computational operations. Indeed, gene circuits performing basic Boolean logic operations in mammalian cells are already available (1–5). However, the creation of more complex gene circuits with sophisticated computational functions remains challenging due to the difficulty of combining multiple core regulation processors into one processing unit. The recently developed Clustered Regularly Interspaced Short Palindromic Repeats/CRISPR-associated protein 9 (CRISPR/Cas9) technology is a highly effective genome-engineering tool for developing synthetic gene circuits (6–12). Here, we used it to develop a CRISPR-based central processing unit (CRISPR-CPU), employing a Cas9-based transcriptional regulation system as a core processor, with synthetic gene circuits utilizing guide RNAs (gRNAs) and corresponding promoters. We then applied this system to generate programmable gene circuits in mammalian cells.

**Fig. 1.**
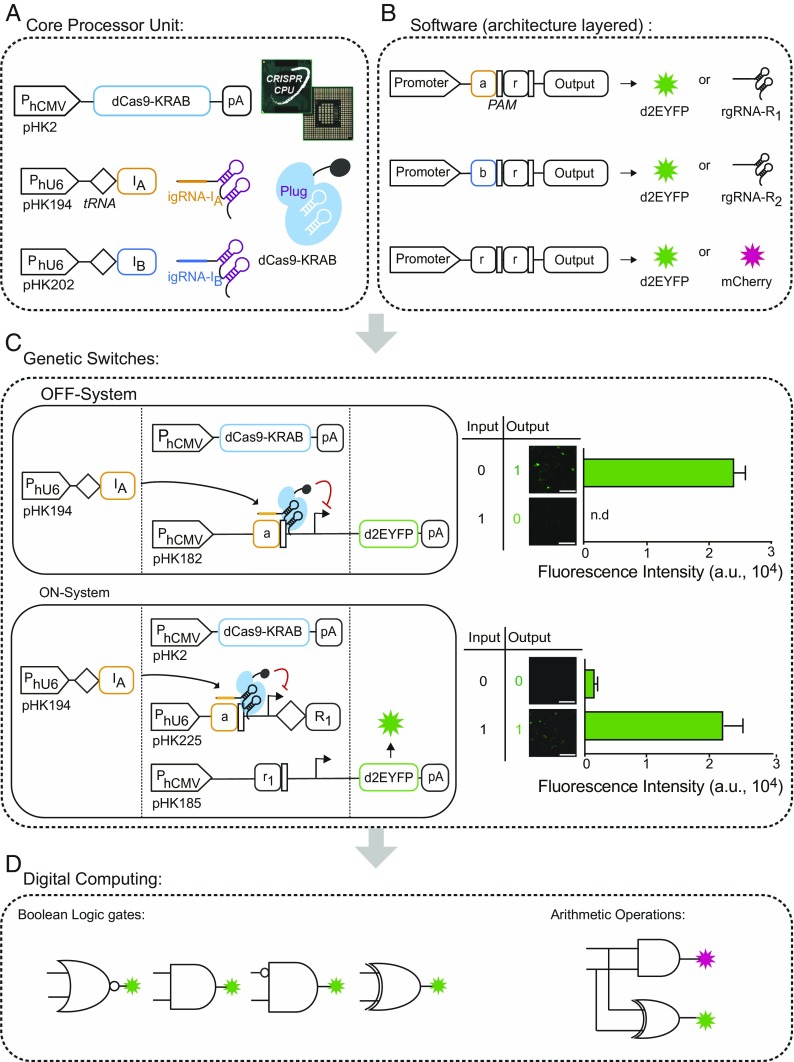
Design of programmable CRISPR-mediated gene switches. (*A*) Genetic components of the core processor. As a transcriptional master regulator, dCas9-KRAB was expressed under the constitutive P_hCMV_ promoter. An igRNA-I (I_A_, orange rectangle; I_B_, light-blue rectangle) was expressed under the constitutive P_hU6_ promoter. tRNA (black rhombus) was processed by intrinsic cellular proteins RNase P and RNaseZ to produce gRNA carrying the desired 5′-target sequences without extra nucleotides. Processed igRNA-I was associated with dCas9 protein. (*B*) Diagram of software architecture layers. Binding sites for igRNA (a or b) and rgRNA (r) with a PAM (black narrow rectangle) sequence between promoter and output were inserted into the output-expressing unit; the output was regulated by the presence of igRNA-I and rgRNA-R. The output can be fluorescent protein, as a final output of the gene circuit, or rgRNA-R as an intermediate regulator of the gene circuit. (*C*) Diagram of OFF/ON system showing transcriptional regulation by the CRISPR system. In the OFF system, a binding site for igRNA(a) between the P_hCMV_ promoter and the reporter gene ORF was inserted into a reporter gene-expressing unit. The igRNA-I_A_ repressed the transcription of reporter genes. In the ON system, a binding site for igRNA-I_A_ between the P_hU6_ promoter and rgRNA-R (r_1_) TSS was inserted into an rgRNA-expressing unit, and a binding site for rgRNA-R_1_ (r_1_) between the P_hCMV_ promoter and reporter gene ORF was inserted into a reporter gene-expressing unit. The igRNA activated the reporter gene transcription by repressing rgRNA-R_1_. Transient transfection of dCas9-KRAB and gRNA expression plasmids repressed reporter gene expression in HEK-293T cells. Cells were transfected with the indicated plasmids for an OFF/ON system (*SI Appendix*, Table S2) and analyzed by flow cytometry for d2EYFP expression at 48-h posttransfection. The data are displayed as the means ± SD of three independent experiments (*n* = 3). Mean fluorescence intensities are presented as arbitrary units (a.u.). (*D*) OFF/ON transcriptional switches were used as building blocks for the digital computing gene circuits, such as Boolean logic gates and the half adder as an arithmetic operator.

One application of the CRISPR/Cas9 system is based on a catalytically inactive form of Cas9 (dead Cas9, dCas9), which functions as a customized DNA-binding protein relying on gRNA sequences to regulate the transcription of a specific target sequence (13). We constructed several transcriptional switches, using gRNA as an input. In the CRISPR-CPU, the Krueppel-associated box protein of the human *kox-1* gene (KRAB) domain, fused to dCas9, served as a transcriptional master repressor (Fig. 1). The repression-based system outperformed dCas9 lacking the KRAB domain and performed as efficiently as the activation-based system (*SI Appendix*, Figs. S1 and S2). Two to four repeats of gRNA binding sequences and the 5′-NGG-3′ protospacer adjacent motif (PAM) were inserted as the “operator,” representing the transcriptional regulatory unit (Fig. 1). For gRNA expression, we adapted an endogenous tRNA-processing system that ensures elimination of the 5′-flanking region sequence of the primary transcript. We designed tRNA-gRNA units by inserting the tRNA sequence between the human U6 (hU6) promoter and the gRNA sequence. The primary transcript is recognized by RNase P and RNase Z, and processed into mature gRNAs carrying the desired 5′-target sequences without extra nucleotides (14). Constructs with tRNA showed comparable activity to that of constructs without tRNA (*SI Appendix*, Fig. S3), and enhanced the performance of the regulatory gRNAs (*SI Appendix*, Fig. S4). This approach was used to build systems with a variety of computational functions, as follows.

The OFF system consisted of three core components, namely dCas9-KRAB, an input gRNA (igRNA), and a reporter construct with binding sites for igRNA between the human cytomegalovirus immediate-early (hCMV) promoter and the transcription start site (TSS). In the OFF system, constitutively expressed dCas9-KRAB inhibited transcription of the reporter gene only in the presence of igRNA (Fig. 1 and *SI Appendix*, Fig. S5). The ON system consisted of four constructs: dCas9-KRAB, an igRNA, a regulatory gRNA (rgRNA) with binding sites for the igRNA between the hU6 promoter and the TSS of the rgRNA, and a reporter construct with binding sites for the rgRNA. In the ON system, rgRNA transcription is blocked by igRNA, so that the reporter gene is turned on only in the presence of igRNA (Fig. 1 and *SI Appendix*, Fig. S6). To test the performance of the ON/OFF switches, we introduced dCas9-KRAB, gRNA, and reporter plasmids into HEK-293T cells and measured fluorescent protein levels at 48 h posttransfection as an output. We observed significant repression and activation in the OFF and ON systems, respectively (Fig. 1 and *SI Appendix*, Figs. S5 and S6). We observed correct circuit performance after 24 h, although the performance was improved after 48 h (*SI Appendix*, Fig. S7). For a single reporter gene controlled by a gRNA, we observed dose-dependent repression (*SI Appendix*, Fig. S8). However, if a cell received the full set of constructs, the designed circuit function was executed and the output was produced in a switch-like manner. Furthermore, fusing a domain for auxin-induced protein degradation to the output protein enabled us to switch off the circuit by the addition of a small molecule (indole-3-acetic acid, IAA). In the absence of auxin, the circuit operated as usual. Addition of auxin shut off the circuit within 4 h, while removal of auxin from the medium restored the circuit function (*SI Appendix*, Fig. S9).

We tested whether the inhibitory effect of rgRNA-R_1_ by igRNA-I_A_ is enhanced by the subsequent introduction of the plasmids. We introduced all ON-system components except rgRNA-R_1_ which was transfected 6 h after the first transfection. We observed that igRNA-I_A_ enabled the reactivation of reporter gene expression by repression of rgRNA-R_1_ via cotransfection. However, a high leakiness was observed in time-delay transfection (*SI Appendix*, Fig. S10). Therefore, we used cotransfection methods for all following experiments.

We chose 4 gRNAs that were highly specific and not subject to intergRNA interference (*SI Appendix*, Fig. S11). CRISPR-mediated transcriptional ON/OFF switches as shown here can be used as building blocks for synthetic gene circuits. The processing unit consists of only one core processor with one master protein (dCas9-KRAB). In principle, such a single processor using orthogonal gRNAs can achieve a capacity of multiple bits.

**Fig. 2.**
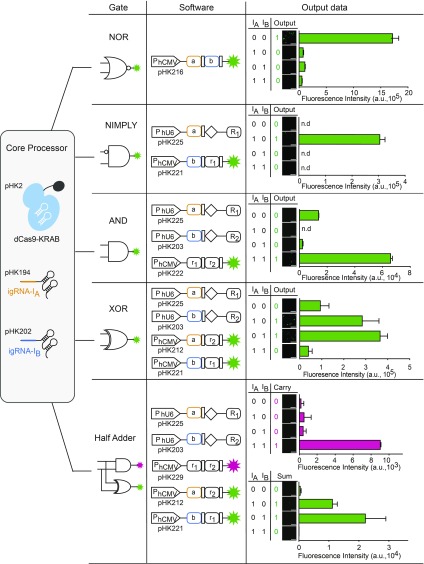
Designing Boolean logic gates (NOR, NIMPLY, and AND) and combinational logic gates (XOR and half adder). Electronic circuit diagram (gate), schematic representation of gene circuit components (software) and the truth table and microscope images and FACS analysis of the performance (output data) of Boolean logic gates and the half adder. All gates have the same core processor but different gene circuit components as software. An A NOR B gate (binding sites for two igRNAs, igRNA-I_A_ and -I_B_), was placed between the P_hCMV_ promoter and the reporter gene. An A NIMPLY B gate (binding site for igRNA-I_B_) was placed between the P_hU6_ promoter and the igRNA rgRNA-R_2_ and binding sites for igRNA-I_A_ and rgRNA-R_2_ were placed between the P_hCMV_ promoter and the reporter gene. An A AND B gate (binding sites for igRNA-I_A_ and -I_B_) was placed between the P_hU6_ promoter and rgRNA-R_1_ and -R_2_ and binding sites for rgRNA-R_1_ and -R_2_ were placed between the P_hCMV_ promoter and the reporter gene. An A XOR B gate (binding sites for igRNA-I_A_ and -I_B_) was placed between the P_hU6_ promoter and rgRNA-R_1_ and - R_2_. Binding sites for igRNA-I_A_ and rgRNA-R_2_ were placed in the reporter construct, and binding sites for igRNA-I_B_ and rgRNA-R_1_ were placed in another reporter construct. Half adder: combining A AND B gate and A XOR B gate for a binary function by calculating the carry (mCherry) and sum (d2EYFP). In accordance with the truth table for each gate and the half adder, transfected HEK-293T cells were programmed to produce d2EYFP or mCherry. Gate performance was confirmed using microscopy and flow cytometry. The data are displayed as the means ± SD for three independent experiments (*n* = 3). Mean fluorescence intensities are presented as a.u.

Next, we designed an A NOR B gate, which is only active in the absence of both igRNAs [**Input (A:0, B:0) = Output (1)**], by combining two OFF systems. Binding sites for two igRNAs, located between the promoter and TSS of the reporter gene, allowed for repression of reporter gene transcription in the presence of either igRNA-I_A_ or -I_B_. This gate provided 15- to 30-fold activation when neither of the two inputs was present (Fig. 2 and *SI Appendix*, Fig. S12). For an A NIMPLY B gate (A ANDNOT B), which is exclusively induced in the presence of only one specific igRNA **[Input (A:1, B:0) = Output (1)]**, we combined one OFF system and one ON system. We generated a reporter plasmid containing binding sites for igRNA-I_A_ and rgRNA-R_1_, which could be repressed by igRNA-I_A_. IgRNA-I_A_ and -I_B_ were able to perform without mutual interference, and rgRNA-R_1_ was found to repress reporter gene transcription in the presence of igRNA-I_A_, but not igRNA-I_B_. The reporter gene could only be transcribed in the presence of igRNA-I_B_ (Fig. 2 and *SI Appendix*, Fig. S13). Next, we built an A AND B gate, which is induced only in the presence of both igRNAs **[Input (A:1, B:1) = Output (1)]**, by combining two ON systems. As igRNA-I_A_ and -I_B_ repressed rgRNA-R_1_ and -R_2_, respectively, induction could be achieved by introducing both igRNAs (Fig. 2 and *SI Appendix*, Fig. S14). Furthermore, we were able to build complex logic functions by combining these simple logic gates. The combination of A NIMPLY B and B NIMPLY A gates generated an A XOR B gate, which integrates two different input signals and produces the output ON only if one of the inputs is present **[Input (A:1, B:0; A:0, B:1)] = Output (1)]**. The circuit consisted of two rgRNAs and two reporters; the rgRNAs were repressed by igRNAs and transcription of the reporter gene was regulated by the combination of igRNAs and rgRNAs. High levels of transcription of the reporter were achieved only when one of the igRNAs was present (Fig. 2 and *SI Appendix*, Fig. S15).

Binary arithmetic is performed by combinational logic gates, of which the simplest is the half adder, which adds two inputs A and B and generates two outputs Carry (C_OUT_) and Sum (S_HA_). The AND gate, which determines the C_OUT_, consisted of an mCherry reporter gene with binding sites for rgRNA-R_1_ and -R_2_. To calculate the S_HA_, the XOR gate was constructed with two d2EYFP-expressing reporters and two rgRNAs, rgRNA-R_1_ and -R_2_, which were used for the AND gates. One of the XOR reporters was repressed by igRNA-I_A_ and rgRNA-R_2_, and the other was repressed by igRNA-I_B_ and rgRNA-R_1_. The combination of A AND B gate and the A XOR B gate enabled cellular half-adder computations, controlled by the presence of igRNAs. In the absence of input, the cell yielded 0 for both outputs **[Input (A:0, B:0)] = Output (C**_**OUT**_**:0, S**_**HA**_**:0)]**. The presence of only one input yielded 0 for C_OUT_ and 1 for S_HA_ **[Input (A:0, B:1; A:1, B:0) = Output (C**_**OUT**_**:0, S**_**HA**_**:1)]**. When both inputs were present, both outputs were 1 **[Input (A:1, B:1)] = Output (C**_**OUT**_**:1, S**_**HA**_**:1)]** (Fig. 2 and *SI Appendix*, Fig. S16).

**Fig. 3.**
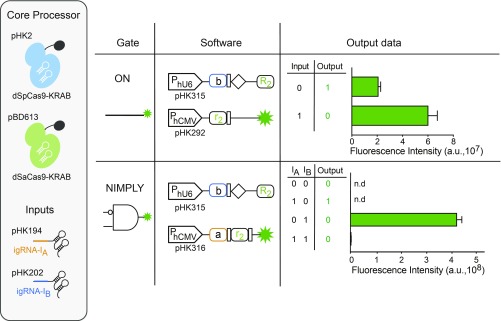
Establishing a dual-core CPU with Boolean logic gate applications. Electronic circuit diagram (gate), schematic representation of the gene circuit components (software), and the truth table and FACS analysis of the performance (output data) of Boolean logic gates. All gates have the same two core processors (dSpCas9-KRAB and dSaCas9-KRAB), but different gene circuit components as software. ON switch: the binding site for igRNA-I_B_ (dSpCas9-KRAB) was placed between the P_hU6_ promoter and rgRNA-R_2_ and the binding site for rgRNA-R_2_ (dSaCas9-KRAB) was placed between the P_hCMV_ promoter and the reporter gene. B NIMPLY A gate: the binding site for igRNA-I_B_ (dSpCas9-KRAB) was placed between the P_hU6_ promoter and rgRNA-R_2_; and binding sites for igRNA-I_A_ and rgRNA-R_2_ (dSaCas9-KRAB) were placed between the P_hCMV_ promoter and the reporter gene. The data are displayed as means ± SD for three independent transfections (*n* = 3). Mean fluorescence intensities are presented as a.u.

We also built dual-core synthetic circuits in single cells by combining two orthogonal CRISPR-based core processors (Fig. 3). In addition to the usual dSpCas9-KRAB, we used *Staphylococcus-aureus*–derived SaCas9 to construct dSaCas9-KRAB as a second, orthogonal computation core, which performed as efficiently as dSpCas9-KRAB (*SI Appendix*, Fig. S17). Recognizing the PAM sequence 5′-NNGRRT-3′, SaCas9 can be used orthogonally with SpCas9. By using dSpCas9-KRAB as the first computation core regulating the software of the second core consisting of dSaCas9-KRAB, we could construct dual-core CPU ON switches (Fig. 3) as well as a NIMPLY gate (Fig. 3) Importantly, the dual-core NIMPLY gate also functioned in immortalized human mesenchymal stem cells (hMSCs), suggesting potential applicability of this system for therapeutic applications (*SI Appendix*, Fig. S18).

One important advantage of this CRISPR-mediated transcriptional regulation system is its efficiency; most transcription-control devices consist of two components: a DNA-binding domain specific for an operator sequence and a transcriptional regulatory domain functioning as a transcriptional activator or repressor (15). To recognize a specific operator sequence, the corresponding DNA-binding protein must physically associate with the DNA operator. However, as dCas9-KRAB serves as the transcriptional master repressor in the present system, its binding specificity is solely reliant on gRNA. Compared with synthetic DNA-binding domains, the generation of gRNA is both cost-effective and user-friendly, and future users can easily modify and extend the existing circuits. In this context, the tRNA-expression system is essential to enable us to build up gene circuits by adding multiple layers of regulation by regulatory gRNAs. For consistency, we equipped all gRNA constructs with the tRNA-processing system, as the compatible structure facilitates the modularity of circuit design. Additionally, as gRNA is itself used as an input signal, the system does not require additional induction. Another advantage of CRISPR-mediated transcriptional regulation is the orthogonality of custom-designed gRNAs and their corresponding promoters (9): Each gRNA-promoter set represents a basic unit for building up circuits, and these units can be easily combined to generate multiple layers of regulation for transcriptional control. The modular nature of CRISPR-CPU should even allow for a predictive in silico design of biocomputing circuits. In the future, circuits with even greater complexity may be achieved by using orthogonal dCas9 (16) or CRISPR-Display (17). Indeed, by adding orthogonal CRISPR-based core processors, we established dual-core synthetic circuits in single cells. Thus, we believe this platform has the potential to introduce multicore processing units, which can be considered as CPUs in the biological-computational realm, into single cells. Furthermore, this technique may be applied to both transiently transfected and endogenous gene circuits (18), providing a potential therapeutic approach for diseases caused by the dysregulation of transcriptional networks (19).

In electronics, adders form the main component of the arithmetic logic unit and therefore are an integral part of processor chips. Generating circuits that can perform an adder function in biological systems is a significant step toward realizing biocomputing systems. For this purpose, the cell may be loaded with custom-programmed circuits, enabling the performance of various functions, as a “biocomputing core.” Each single cell can be considered a single-bit core, and multicore bioprocessing with millions or billions of cells may have even more potential for scaling than electronic computing systems. Such biocomputing systems have enormous potential for diagnostic, therapeutic, and biotechnological applications.

For biomedical application of synthetic circuits, a device could be developed to detect specific biomarkers as input signals for an inducible igRNA expression system, to process the information, and then to produce therapeutic outputs (1, 15). Therapeutic applications of programmed synthetic circuits in mammalian cell systems have already been reported (20, 21). Moreover, the half-adder circuit can be used for the detection of two different specific biomarkers as inputs and can produce two different outputs, one for a sentinel function and one for a therapeutic function, depending on the combination of inputs. For example, if only one biomarker is active, the system could activate the sentinel output function, while when two inputs are active, it could produce a functional therapeutic output. Thanks to the characteristics of the CRISPR/Cas9 system, these outputs could even be endogenous genes. Such a versatile calculator could, for instance, be used in biomedical research for highly specific integration of multiple disease-relevant inputs and the subsequent output of an effector protein as well as a signaling output for a positive bystander effect. Future research incorporating inducible gRNAs or inducible dCas9-variants into the CRISPR-CPU and combining several CRISPR-CPUs from orthogonal Cas9-variants will enable substantial progress in the field of biocomputing. Using these modular building blocks, the construction of a full adder or even more sophisticated gene circuits with the CRISPR-CPU technology might be one of the future stepping stones for mammalian synthetic biology.

In summary, we present a programming architecture, using the CRISPR/Cas9 technology, in which a single dCas9-KRAB transcriptional repressor functions as the core processor that can be programmed to perform complex operations with different sets of user-defined gRNA inputs. This repression-based system is superior to activation-based systems, since all Boolean logic gates can be built with a very low metabolic load. Using the CRISPR-CPU, we demonstrate complex applications of a master repression unit controlling multiple gRNA expression levels. Using combinations of transcriptional regulation switches, we were able to build various gene circuits, including a circuit that performed binary arithmetic. We believe the CRISPR-CPU provides a user-friendly programming interface with the potential to provide large-scale biocomputational capacity.

## Methods

### Plasmid Construction.

Comprehensive design and construction details for all expression vectors are provided in *SI Appendix*, Table S1.

### Cell Culture and Transfection.

Human embryonic kidney (HEK) cells from the 293T cell line American Type Culture Collection (ATCC) no. CRL-11268 and human telomerase reverse transcriptase-immortalized human mesenchymal stem cells (hMSC-hTERT, ATCC: SCRC-4000) were cultured in DMEM (Invitrogen) supplemented with 10% heat-inactivated FBS (Invitrogen) and 1% penicillin/streptomycin (Biowest) at 37 °C in a 5% CO_2_ environment. 1.0 × 10^5^/4.0 × 10^5^ cells per well were plated in 500 µL/2 mL of media in a 24-well/6-well plate. A total plasmid mass of 1–4 µg per well was transfected using 3–12 µL per well of polyethyleneimine (1 mg/mL). At 24 h after plating, cells were transfected with plasmid DNA as indicated in *SI Appendix*, Tables S2 and S4. The plasmid ratio and amount transfected are provided in *SI Appendix*, Tables S2 and S4.

### Luciferase Reporter Gene Assay.

The expression levels of P_*HER2*_-driven luciferase reporter were assessed 48 h posttransfection. The luciferase profiling was performed according to the following protocol. Initially, growth medium was removed from all of the wells. Subsequently, 60 μL of 1 × Lysis reagent (E1531, Promega) was added. After observing cell detachment, 200 μL of Luciferase Assay Reagent was supplemented. Luciferase Assay Reagent was prepared by 10× dilution of 5 mM d-luciferin (potassium salt) solution (Art.-Nr. M03620-250MG, Chemie Brunschwig AG) together with complete DMEM. Immediately, 2 × 125 μL were transferred to two separate wells on 96-well black plate (µClear-Boden, Art.-Nr. 7.655 090, Greiner Bio One). The luminescence signal was quantified using an Envision 2104 multilabel plate reader (Perkin-Elmer).

### Flow Cytometry.

Cells were washed twice with ice-cold PBS and harvested with 200 μL of FACS buffer (1% BSA and 0.5 mM EDTA in PBS) 3 d after transfection. Flow cytometry analysis was performed using a BD FACS Fortessa flow cytometer. A 488-nm diode laser was used for the detection of d2EYFP, a 688-nm diode laser was used for the detection of mCherry, and a 633-nm diode laser was used to detect the iRFP transfection control. In each sample, viable singlet HEK-293T cells were gated via forward-scatter laser and side-scatter and at least 10,000 cells were analyzed as iRFP-positive cells (*SI Appendix*, Fig. S19). The collected data were analyzed using FlowJo (TreeStar) software. The data represent the results of at least two independent experiments.

### Fluorescence Imaging.

Fluorescence and time-lapse microscopy was performed with an inverted fluorescence microscope (Nikon Ti-E, Nikon) equipped with an incubation chamber, an Orca Flash 4 digital camera [Hammamatsu a pE-100-LED (CoolLED)] as the transmission-light source, a Spectra X (Lumencor) as the fluorescent-light source and a 10× objective (Plan Apo λ; numerical aperture, 0.45; DIC N1; working distance, 4). Bright-field images (3% intensity, 90-ms exposure), d2EYFP fluorescence images (excitation, 513/17 nm; intensity, 50%; exposure, 200 ms; YFP ET filter, dichroic 520 nm; emission, 543/22 nm), and mCherry fluorescence images (excitation, 549/15 nm; intensity, 50%; exposure, 200 ms; CY3 HC, dichroic 562 nm; emission, 593/40) were collected. A binning of 2 × 2 was used.

### Auxin-Induced Degron Experiments.

DNA was transfected into 2.5 × 10^5^ HEK-293T cells seeded the day before in a 24-well plate. After 48 h, fluorescence was measured in one set of wells and 500 µM IAA, in ethanol, SigmaAldrich I3750-5G-A) were added to the remaining wells. Four hours later, fluorescence was measured in the next set of wells. The last set of wells was washed twice with normal DMEM, the medium was exchanged to DMEM, and 6 h later, the fluorescence in these wells was measured.

## Supplementary Material

Supplementary File
