# Supplementary material for: A CRISPR/Cas9-based central processing unit to program complex logic computation in human cells
Source: Proc Natl Acad Sci U S A. 2019 Mar 28;116(15):7214–9. doi: 10.1073/pnas.1821740116 (PMC6462112; doi:10.1073/pnas.1821740116)
Supplement: Supplementary File [file pnas.1821740116.sapp.pdf]

Supplementary Information

A CRISPR/Cas9-based *C*entral *P*rocessing *U*nit to program complex logic computations in human cells

Hyojin Kim[a,1,2], Daniel Bojar[a,2] and Martin Fussenegger[a,b,3]

[a]Department of Biosystems Science and Engineering, ETH Zurich, Mattenstrasse 26, CH-4058 Basel, Switzerland; [b]Faculty of Science, University of Basel, Mattenstrasse 26, CH-4058 Basel, Switzerland;

[1]Present address: The Breast Cancer Now Toby Robins Research Centre, Institute of Cancer Research, Fulham Road, London SW3 6JB, UK

[2]H.K. and D.B. contributed equally to this work.

[3]To whom correspondence should be addressed: Tel.: +41 61 387 31 60, Fax: +41 61 387 39 88, E-mail: fussenegger@bsse.ethz.ch

www.pnas.org/cgi/doi/10.1073/pnas.1821740116

Supplementary Results
Supplementary Tables
Supplementary Table 1. Plasmids designed and used in this study

Plasmid	Description and Cloning Strategy	Reference or Source
pcDNA3.1(+)	Constitutive mammalian expression vector (P_{hCMV}-MCS-pA).	Life Technologies
pEGFP-N1	Constitutive mammalian EGFP expression vector (P_{hCMV}-EGFP-pA).	Clontech
pd2EYFP-N1	Constitutive mammalian d2EYFP expression vector (P_{hCMV}-d2EYFP-pA).	Clontech
pmCherry	Constitutive mammalian mCherry expression vector (P_{hCMV}-mCherry-pA).	Clontech
pMM581	Constitutive mammalian iRFP expression vector (P_{hCMV}-iRFP670-pA).	Müller *et al.*[1]
pNeuLite	Vector encoding luciferase reporter gene expression driven by the HER-2/*neu* promoter (P_{hHER2}-Luciferase-pA).	Addgene #16247
pdSpCas9-humanized	Constitutive mammalian dCas9 expression vector (P_{hCMV}-dSpCas9-pA).	Addgene #44246
pdSpCas9-KRAB/pHK2	Constitutive mammalian dSpCas9-KRAB expression vector (P_{hCMV}-dSpCas9-KRAB-pA).	This work
pU6-SpgRNA	Constitutive mammalian P_{hU6}-driven SpgRNA expression vector containing scaffold region of SpgRNA (P_{hU6}-empty SpgRNA).	This work
pMK294	Vector encoding mAID and mCherry expression cassette (mAID-mCherry2-Bsr-pA).	Addgene #72832
pMK232	Vector encoding OsTIR1 expression cassette (P_{hCMV}-OsTIR1-puro).	Addgene #72834
AAV_NLS-dSaCas9-NLS-VPR	Constitutive mammalian pdSaCas9-VPR expression vector (P_{hCMV}-dSaCas9-VPR-pA).	Addgene #68495
pSaGuide	Constitutive mammalian P_{hU6}-driven SagRNA expression vector containing scaffold region of SagRNA (P_{hU6}-empty SagRNA).	Addgene #64710
pWH5	Vector encoding $P_{SV40\text{-}2xoPmeR}$-driven SEAP expression cassette ($P_{SV40\text{-}2xoPmeR}$-SEAP).	Wang *et al.*[2]
pJP4	Vector encoding constitutive expression of igRNA-I_A for dSpCas9-KRAB (P_{hU6}-SpgRNA-I_A). The oligonucleotides oJP43 (5'-ccggCCAATCACAGGAG AAGGAGG-3') and oJP44 (5'-aaacCCTCCTTCTCCTGTGATTGG-3') for the target sequence of SpgRNA-I_A were annealed and cloned into *Bsa*I-restricted pU6-SpgRNA.	This work
pJP47	Vector encoding constitutive expression of rgRNA-R_1 for dSpCas9-KRAB (P_{hU6}-SpgRNA-R_1). The oligonucleotides oJP53 (5'-ccggCGTGCGAATCTGG TGGCACC-3') and oJP54 (5'-aaacGGTGCCACCAGATTCGCACG-3') for the target sequence of SpgRNA-R_1 were annealed and cloned into *Bsa*I-restricted pU6-SpgRNA.	This work
pJP52	Vector encoding constitutive expression of rgRNA-R_2 for dSpCas9-KRAB (P_{hU6}-SpgRNA-R_2). The oligonucleotides oJP63 (5'-ccggTCTGTTGTGTGAC TCTGGTA-3') and oJP64 (5'-aaacTACCAGAGTCACACAACAGA-3') for the target sequence of SpgRNA-R_2 were annealed and cloned into *Bsa*I-restricted pU6-SpgRNA.	This work

pHK56	Vector encoding constitutive expression of igRNA-I$_B$ for dSpCas9-KRAB (P$_{hU6}$-SpgRNA-I$_B$). The oligonucleotides oHK209 (5’-ccggGCTGACAACCT GCAGCACCA-3’) and oHK210 (5’-aaacTGGTGCTGCAGGTTGTCAGC-3’) for the target sequence of SpgRNA-I$_B$ were annealed and cloned into *Bsa*I-restricted pU6-SpgRNA.	This work
pHK62	Vector encoding P$_{hCMV}$-driven d2EYFP expression unit containing binding sites of SpgRNA-I$_B$ downstream of P$_{hCMV}$ (P$_{hCMV}$-b-d2EYFP-pA). The oligonucleotides oHK221 (5’-ctagCGCTGACAACCTGCAGCACCACGG GCTGACAACCTGCAGCACCACGGGCTGACAACCTGCAGCACCAC GGGCTGACAACCTGCAGCACCACGGA-3’) and oHK222 (5’-agctT CCGTGGTGCTGCAGGTTGTCAGCCCGTGGTGCTGCAGGTTGTCA GCCCGTGGTGCTGCAGGTTGTCAGCCCGTGGTGCTGCAGGTTGT CAGCG-3’) for the binding sequences of SpgRNA-I$_B$ were annealed and cloned into *Nhe*I/*Hin*dIII-restricted pEGFP-N1. D2EYFP was PCR-amplified from pd2EYFP using oligonucleotides oHK182 (5’-gcgggatccGCCACCATGG TGAGCAAGGGCGAGGA-3’) and oHK183 (5’-ggcgcggccgcCTACACA TTGATCCTAGCAG-3’), restricted with *Bam*HI/*Not*I, and cloned into the corresponding sites (*Bam*HI/*Not*I) of the same vector.	This work
pHK63	Vector encoding P$_{hCMV}$-driven d2EYFP expression unit containing binding sites of SpgRNA-R$_3$ downstream of P$_{hCMV}$. The oligonucleotides oHK223 (5’-tcgaCCATTGGACACAGAATCCGAGTGGCATTGGACACAGAATCCG AGTGGCATTGGACACAGAATCCGAGTGGCATTGGACACAGAATC CGAGTGGG-3’) and oHK224 (5’-gatcCCCACTCGGATTCTGTGTCCA ATGCCACTCGGATTCTGTGTCCAATGCCACTCGGATTCTGTGTCC AATGCCACTCGGATTCTGTGTCCAATGG-3’) for the binding sequences of SpgRNA were annealed and cloned into *Sal*I/*Bam*HI-restricted pEGFP-N1. D2EYFP was PCR-amplified from pd2EYFP using oligonucleotides oHK182 (5’-gcgggatccGCCACCATGGTGAGCAAGGGCGAGGA-3’) and oHK183 (5’-ggcgcggccgcCTACACATTGATCCTAGCAG-3’), restricted with *Bam*HI/*Not*I, and cloned into the corresponding sites (*Bam*HI/*Not*I) of the same vector.	
pHK75	Vector encoding SpgRNA-R$_3$ expression unit containing binding sites of SpgRNA-I$_C$ downstream of P$_{hU6}$ (P$_{hU6}$-I$_C$-SpgRNA-R$_3$). The oligonucleotides oHK240 (5’-ccggTAACCTGCTGCAGCAGCCGCGCGGAACCTGCTGC AGCAGCCGCGCGGGCTGACAACCTGCAGCACCACGGGCTGACA ACCTGCAGCACCACGGG-3’) and oHK241 (5’-gatcCCCGTGGTGCTG CAGGTTGTCAGCCCGTGGTGCTGCAGGTTGTCAGCCCGCGCGGC TGCTGCAGCAGGTTCCGCGCGGCTGCTGCAGCAGGTTA-3’) for the binding sequences of SpgRNA-I$_C$ were annealed and cloned into *Age*I/*Bam*HI-restricted pU6-SpgRNA (pHK72). The oligonucleotides oHK236 (5’-gatcCCATTGGACACAGAATCCGAG-3’) and oHK237 (5’-aaacCTCG GATTCTGTGTCCAATGG-3’) for the SpgRNA-R$_3$ expression unit were annealed and cloned into *Bam*HI/*Bsa*I-restricted pHK72.	This work
pHK76	Vector encoding a regulatory SpgRNA-R$_3$ expression unit containing binding sites of P$_{hU6}$ (P$_{hU6}$-d-SpgRNA-R$_3$). The oligonucleotides oHK255 (5’-ccggTCGTCTTCTCCAGGATCACAACGGCGTCTTCTCCAGGATCAC AACGGGCTGACAACCTGCAGCACCACGGGCTGACAACCTGCAGC	This work

	**ACCA***CGG*G-3') and oHK243 (5'-gatcC*CCG***TGGTGCTGCAGGTTGTC AGC***CCG***TGGTGCTGCAGGTTGTCAGC***CCG***TTGTGATCCTGGAGA AGACG***CCG***TTGTGATCCTGGAGAAGACG**A-3') for the binding sequences of SpgRNA-I_D were annealed and cloned into *Age*I/*Bam*HI-restricted pHK75.	
pHK136	Vector encoding P_{hU6}-driven tRNA and scaffold region of SpgRNA expression vector (P_{hU6}-tRNA-empty SpgRNA). The oligonucleotides oHK300 (5'-ccggG GATCCAACAAAGCACCAGTGGTCTAGTGGTAGAATAGTACCCTGTT ATGGTACAGACCCGGGTTCGATTCCCGGCTGGTGCAGAGACCGCTA GCGGTCTCC-3') and oHK301 (5'-aaacGGAGACCGCTAGCGGTCTCTGC ACCAGCCGGGAATCGAACCCGGGTCTGTACCATAACAGGGTACTAT TCTACCACTAGACCACTGGTGCTTTGTTGGATCC-3') for the tRNA sequences were annealed and cloned into *Bsa*I-restricted pU6-SpgRNA.	This work
pHK140	Vector encoding a regulatory SpgRNA-R_3 expression unit with tRNA downstream of P_{hU6} (P_{hU6}-tRNA-SpgRNA-R_3). The oligonucleotides oHK312 (5'-ccggT**GCTGACAACCTGCAGCACCA***CGG***GCTGACAACCTGCAG CACCA***CGG*G-3') and oHK313 (5'-gatcC*CCG***TGGTGCTGCAGGTTGT CAGC***CCG***TGGTGCTGCAGGTGTCAGC**G-3') for the expression unit of SpgRNA-R_3 were annealed and cloned into *Bsa*I-restricted pHK136.	
pHK141	Vector encoding a regulatory SpgRNA-R_3 expression unit containing binding sites of SpgRNA-I_C with tRNA downstream of P_{hU6} (P_{hU6}-c-tRNA-SpgRNA-R_3). tRNA-SpgRNA-R_3 expression cassette were restricted with *Bam*HI/*Xba*I from pHK140 and cloned into corresponding sites (*Bam*HI/*Xba*I) of pHK75.	This work
pHK142	Vector encoding a regulatory SpgRNA-R_3 expression unit containing binding sites of SpgRNA-I_D with tRNA downstream of P_{hU6} (P_{hU6}-d-tRNA-SpgRNA-R_3). tRNA-SpgRNA-R_3 expression cassette were restricted with *Bam*HI/*Xba*I from pHK140 and cloned into corresponding sites (*Bam*HI/*Xba*I) of pHK76.	This work
pHK182	Vector encoding P_{hCMV}-driven d2EYFP expression unit containing binding sites of igRNA-I_A downstream of P_{hCMV} (P_{hCMV}-a-d2EYFP-pA). The oligonucleotides oHK349 (5'-ctagC**CCAATCACAGGAGAAGGAGG***AGG* **CCAATCACAGGAGAAGGAGG***AGG*G-3') and oHK350 (5'-aattC*CCT***CC TCCTTCTCCTGTGATTGG***CCT***CCTCCTTCTCCTGTGATTGG**G-3') for the binding sequences of igRNA-I_A were annealed and cloned into *Nhe*I/*Eco*RI-restricted pd2EYFP-N1.	This work
pHK185	Vector encoding P_{hCMV}-driven d2EYFP expression unit containing binding sites of rgRNA-R_1 downstream of P_{hCMV} (P_{hCMV}-r_1-d2EYFP-pA). The oligonucleotides oHK355 (5'-ctagC**CGTGCGAATCTGGTGGCACC***AGG* **CGTGCGAATCTGGTGGCACC***AGG*G-3') and oHK356 (5'-aattC**GGTG CCACCAGATTCGCACG***CCT***GGTGCCACCAGATTCGCACG***CCT*G-3') for the binding sequences of rgRNA-R_1 were annealed and cloned into *Nhe*I/*Eco*RI-restricted pd2EYFP-N1.	This work
pHK189	Vector encoding P_{hCMV}-driven d2EYFP expression unit containing binding sites of rgRNA-R_2 downstream of P_{hCMV} (P_{hCMV}-r_2-d2EYFP-pA). The oligonucleotides oHK363 (5'-ctagC**TCTGTTGTGTGACTCTGGTA***AGG***T CTGTTGTGTGACTCTGGTA***AGG*G-3') and oHK364 (5'-aaac**TACCAG AGTCACACAACAGA***CCT***TACCAGAGTCACACAACAGA**G-3') for the	This work

	binding sequences of rgRNA-R_2 were annealed and cloned into *Nhe*I/*Eco*RI-restricted pd2EYFP-N1.	
pHK194	Vector encoding constitutive expression of igRNA-I_A with tRNA downstream of P_{hU6} (P_{hU6}-tRNA-SpgRNA-I_A). The oligonucleotides oHK371 (5’-gtgc**CCAATCACAGGAGAAGGAGG**-3’) and oJP44 (5’-**aaacCCTCCTTCTCCTGTGATTGG**-3’) for the target sequence of igRNA-I_A were annealed and cloned into *Bsa*I-restricted pHK136.	This work
pHK197	Vector encoding constitutive expression of rgRNA-R_1 with tRNA downstream of P_{hU6} (P_{hU6}-tRNA-SpgRNA-R_1). The oligonucleotides oHK374 (5’-gtgc**CGTGCGAATCTGGTGGCACC**-3’) and oJP54 (5’-**aaacGGTGCCACCAGATTCGCACG**-3’) for the target sequence of rgRNA-R_1 were annealed and cloned into *Bsa*I-restricted pHK136.	This work
pHK201	Vector encoding constitutive expression of rgRNA-R_2 with tRNA downstream of P_{hU6} (P_{hU6}-tRNA-SpgRNA-R_2). The oligonucleotides oHK378 (5’-gtgc**TCTGTTGTGTGACTCTGGTA**-3’) and oJP64 (5’-**aaacTACCAGAGTCACACAACAGA**-3’) for the target sequence of rgRNA-R_2 were annealed and cloned into *Bsa*I-restricted pHK136.	This work
pHK202	Vector encoding constitutive expression of igRNA-I_B with tRNA downstream of P_{hU6} (P_{hU6}-tRNA-SpgRNA-I_B). The oligonucleotides oHK379 (5’-gtgc**GCTGACAACCTGCAGCACCA**-3’) and oHK210 (5’-**aaacTGGTGCTGCAGGTTGTCAGC**-3’) for the target sequence of igRNA-I_B were annealed and cloned into *Bsa*I-restricted pHK136.	This work
pHK203	Vector encoding a regulatory rgRNA-R_2 expression unit containing binding sites of igRNA-I_B with tRNA downstream of P_{hU6} (P_{hU6}-b-tRNA-gRNA-R_2). The oligonucleotides oHK380 (5’-ccggTGCTGACAACCTGCAGCACCACGGGCTGACAACCTGCAGCACCACGGG-3’) and oHK381 (5’-gatcCCCGTGGTGCTGCAGGTTGTCAGCCCGTGGTGCTGCAGGTGTCAGCA-3’) for the binding sequences of igRNA-I_B were annealed and cloned into *Age*I/*Bam*HI-restricted pU6-SpgRNA (pHK2031). RgRNA-R_2 expression cassette were restricted with *Bam*HI/*Xba*I from pHK201 and cloned into corresponding sites (*Bam*HI/*Xba*I) of pHK2031.	This work
pHK212	Vector encoding P_{hCMV}-driven d2EYFP expression unit containing binding sites of igRNA-I_A and rgRNA-R_2 downstream of P_{hCMV} (P_{hCMV}-a-r_2-d2EYFP-pA). The oligonucleotides oHK398 (5’-ctagCCCAATCACAGGAGAAGGAGGAGGCCAATCACAGGAGAAGGAGGAGGTCTGTTGTGTGACTCTGGTAAGGTCTGTTGTGTGACTCTGGTAAGGG-3’) and oHK-399 (5’-aattCCCTTACCAGAGTCACACAACAGACCTTACCAGAGTCACACAACAGACCTCCTCCTTCTCCTGTGATTGGCCTCCTCCTTCTCCTGTGATTGGG-3’) for the binding sequences of igRNA-I_A and rgRNA-R_2 were annealed and cloned into *Nhe*I/*Eco*RI-restricted pd2EYFP-N1.	This work
pHK216	Vector encoding P_{hCMV}-driven d2EYFP expression unit containing binding sites of igRNA-I_A and -I_B downstream of P_{hCMV} (P_{hCMV}-a-b-d2EYFP-pA). The oligonucleotides oHK406 (5’-aattCGCTGACAACCTGCAGCACCACGGGCTGACAACCTGCAGCACCACGGCCAATCACAGGAGAAGGAGGAGGCCAATCACAGGAGAAGGAGGAGGG-3’) and oHK407 (5’-gatcCCCTCCTCCTTCTCCTGTGATTGGCCTCCTCCTTCTCCTGTGATTGG	This work

	**CCGTGGTGCTGCAGGTTGTCAGCCCGTGGTGCTGCAGGTTGTC AGC**G-3') for the binding sequences of igRNA-I_A and -I_B were annealed and cloned into _Eco_RI/_Bam_HI-restricted pd2EYFP-N1.	
pHK221	Vector encoding P_{hCMV}-driven d2EYFP expression unit containing binding sites of igRNA-I_B, and rgRNA-R_1 downstream of P_{hCMV} (P_{hCMV}-b-r_1-d2EYFP-pA). The oligonucleotides oHK412 (5'-aattC**GCTGACAACCT GC AGCACCA**_CGG_**GCTGACAACCTGCAGCACCA**_CGG_G-3') and oHK413 (5'-gatcC_CCG_**TGGTGCTGCAGGTTGTCAGC**_CCG_**TGGTGCTGCAGG TTGTCAGC**G-3') for the binding sequences of igRNA-I_B were annealed and cloned into _Eco_RI/_Bam_HI-restricted pHK185.	This work
pHK222	Vector encoding P_{hCMV}-driven d2EYFP expression unit containing binding sites of rgRNA-R_1 and -R_2 downstream of P_{hCMV} (P_{hCMV}-r_1-r_2-d2EYFP-pA). The oligonucleotides oHK414 (5'-aattC**TCTGTTGTGTGACTCTGGTA** _A GG_**TCTGTTGTGTGACTCTGGTA**_AGG_G-3') and oHK415 (5'-gatcC_CC T_**TACCAGAGTCACACAACAGA**_CCT_**TACCAGAGTCACACAACAGA** G-3') for the binding sequences of rgRNA-R_2 were annealed and cloned into _Eco_RI/_Bam_HI-restricted pHK185.	This work
pHK225	Vector encoding a regulatory rgRNA-R_1 expression unit containing binding sites of igRNA-I_A with tRNA downstream of P_{hU6} (P_{hU6}-a-tRNA-SpgRNA-R_1). The oligonucleotides oHK382 (5'-ccggT**CCAATCACAGGAGAAGGAGG**_A GG_**CCAATCACAGGAGAAGGAGG**_AGG_G-3') and oHK383 (5'-gatcC_CC T_**CCTCCTTCTCCTGTGATTGG**_CCT_**CCTCCTTCTCCTGTGATT GG**G-3') for the binding sequences of igRNA-I_A were annealed and cloned into _Age_I/_Bam_HI-restricted pU6-SpgRNA (pHK2251). RgRNA-R_1 expression cassette was restricted with _Bam_HI/_Xba_I from pHK197 and cloned into corresponding sites (_Bam_HI/_Xba_I) of pHK2251.	This work
pHK229	Vector encoding P_{hCMV}-driven mCherry expression unit containing binding sites of rgRNA-R_1 and -R_2 downstream of P_{hCMV} (P_{hCMV}-r_1-r_2-mCherry-pA). The mCherry was digested by _Bam_HI/ _Not_I from pmCherry vector and cloned into _Bam_HI/_Not_I-restricted pHK222.	This work
pHK277	Vector encoding P_{hU6}-driven tRNA and scaffold region of SagRNA expression vector (P_{hU6}-tRNA-empty SagRNA). The oligonucleotides oHK477 (5'-caccACGCGTGACGTCACCGGTaacaaagcaccagtggtctagtggtagaatagtaccctgttat ggtacagacccgggttcgattcccggctggggtcttcgagaagacct-3') and oHK478 (5'-aaac aggtcttctcgaagaccccagccgggaatcgaacccgggtctgtaccataacagggtactattctaccactagac cactggtgctttgttACCGGTGACGTCACGCGT-3') were annealed for the tRNA sequences and cloned into _Bbs_I-restricted pSaGuide vector.	This work
pHK284	Vector encoding a regulatory rgRNA-R_1 expression unit for dSaCas9 with tRNA downstream of P_{hU6} (P_{hU6}-tRNA-SagRNA-R_1). The oligonucleotides oHK485 (5'-gctg**CGTGCGAATCTGGT**-3') and oHK489 (5'-aaac**ACCAGATTC GCACG**-3') were annealed for the SagRNA-R_1 sequences and cloned into _Bbs_I-restricted pHK277.	This work
pHK285	Vector encoding a regulatory rgRNA-R_2 expression unit for dSaCas9 with tRNA downstream of P_{hU6} (P_{hU6}-tRNA-SagRNA-R_2). The oligonucleotides oHK486 (5'-gctg**TCTGTTGTGTGACT**-3') and oHK490 (5'-aaac**AGTCACACAAC**	This work

	**AGA**-3') were annealed for the SagRNA-R_2 sequences and cloned into *Bbs*I-restricted pHK277.	
pHK292	Vector encoding P_{hCMV}-driven EGFP expression unit containing binding sites of rgRNA-R_2 downstream of P_{hCMV} (P_{hCMV}-r_2-EGFP-pA). Anealing oligos oHK501 (5'-aattC**TCTGTTGTGTGACT**CC*GAAT***TCTGTTGTGTGACT**CC*GAAT*G-3') and oHK502 (5'-gatcC*ATTCGG***AGTCACACAACAGA***ATTCGG***AGTCACACAACAGA**G-3') and cloned into *Eco*RI/*Bam*HI digested pd2EYFP-N1.	This work
pHK310	Vector encoding P_{hCMV}-driven miniAID-d2EYFP expression unit containing binding sites of igRNA-I_A downstream of P_{hCMV} (P_{hCMV}-a-miniAID-d2EYFP-pA). MiniAID was PCR-amplified from pMK294 (mAID-mCherry2-Bsr) using oHK511 (5'-ggcgaattcAAGGAGAAGAGTGCTTGTCC-3') and oHK512 (5'-ggcggatccTTTATACATCCTCAAATCGA-3'), restricted with *Eco*RI/*Bam*HI and cloned into the corresponding sites (*Eco*RI/*Bam*HI) of pHK182.	This work
pHK311	Vector encoding P_{hCMV}-driven miniAID-d2EYFP expression unit containing binding sites of rgRNA-R_1 downstream of P_{hCMV} (P_{hCMV}-r_1-miniAID-d2EYFP-pA). MiniAID was PCR-amplified from pMK294 (mAID-mCherry2-Bsr) using oHK511 (5'-ggcgaattcAAGGAGAAGAGTGCTTGTCC-3') and oHK512 (5'-ggcggatccTTTATACATCCTCAAATCGA-3'), restricted with *Eco*RI/*Bam*HI and cloned into the corresponding sites (*Eco*RI/*Bam*HI) of pHK185.	This work
pHK312	Vector encoding P_{hCMV}-driven miniAID-d2EYFP expression unit containing binding sites of igRNA-I_B, and rgRNA-R_1 downstream of P_{hCMV} (P_{hCMV}-b-r_1-miniAID-d2EYFP-pA). MiniAID was PCR-amplified from pMK294 (mAID-mCherry2-Bsr) using oHK511 (5'-ggcgaattcAAGGAGAAGAGTGCTTGTC C-3') and oHK512 (5'-ggcggatccTTTATACATCCTCAAATCGA-3'), restricted with *Eco*RI/*Bam*HI and cloned into the corresponding sites (*Eco*RI/*Bam*HI) of pHK221.	This work
pHK315	Vector encoding a regulatory SagRNA-R_2 expression unit containing binding sites of igRNA-I_B with tRNA downstream of P_{hU6} (P_{hU6}-b-tRNA-SpgRNA-R_2). The oligonucleotides oHK517 (5'-cgcgT**GCTGACAACCTGCA***CGG***GCTGACAACCTGCA***CGG*A-3') and oHK518 (5'-ccggT*CCG***TGCAGGTTGTCAGC***CCG***TGCAGGTTGTCAGC**A-3') were annealed for SpgRNA-I_B and cloned into *Mlu*I/*Age*I-restricted pHK285.	This work
pHK316	Vector encoding P_{hCMV}-driven d2EYFP expression unit containing binding sites of igRNA-I_A and SagRNA-R_2 (P_{hCMV}-a-r_2-d2EYFP-pA). The oligonucleotides oHK349 (5'-ctagC**CCAATCACAGGAGAAGGAGG***AGG***CCAATCACAGGAGAAGGAGG***AGG*G-3') and oHK350 (5'-aattC*CCT***CCTCCTTCTCCTGTGATTGG***CCT***CCTCCTTCTCCTGTGATTGG**G-3') were annealed and cloned into *Nhe*I/*Eco*RI-digested pHK292.	This work
pDB219	Vector encoding $P_{2xtetO\text{-}hCMVmin}$-driven secTEV-CALR expression unit. SecTEV-CALR was obtained by digesting pDB198 (P_{hCMV}-secTEV-CALR) with *Nhe*I/*Xba*I and inserted into *Nhe*I/*Xba*I-digested pRK152 ($P_{2xtetO\text{-}hCMVmin}$-nluc).	This work
pDB234	Vector encoding P_{hCMV}-driven EYFP expression unit (P_{hCMV}-EYFP-pA). The EYFP domain was PCR-amplified from pEYFP-C1 using oDB372 (5'-tatgaattcGCCACCATGGTGAGCAAGGGCGAGGAGC-3') and oDB373 (5'-tattctagattaCTTGTACAGCTCGTCCATGCCGAG-3'), digested with *Eco*RI/ *Xba*I and inserted into *Eco*RI/*Xba*I digested pWH5.	This work

pDB613	Vector encoding P_{hCMV}-driven dSaCas9-KRAB expression unit (P_{hCMV}-dSaCas9-pA). The KRAB domain was PCR-amplified from pHK2 using oDB916 (5'-tatggccggccaggcaaaaaagaaaaagGGCGGCTCCGGTGAAAAAGC-3') and oDB917 (5'-taagaattcTCAAACTGATGATTTGATTTCAAATGCAGTC-3'), digested with *Fse*I/*Eco*RI and cloned into the corresponding sites (*Fse*I/*Eco*RI*)* of AAV_NLS-dSaCas9-NLS-VPR (Addgene #68495).	This work
pDB615	Vector encoding P_{hU6}-driven igRNA-I_A for dSaCas9 (P_{hU6}-SagRNA-I_A). oDB919 (5'-caccC**CAATCACAGGAGAAGGAGG**-3') and oDB920 (5'-aaac**CCTCCTTCTCCTGTGATTG**G-3') were annealed and inserted into *Bbs*I-digested pSaGuide (Addgene # 64710).	This work
pDB616	Vector encoding P_{hCMV}-driven d2EYFP expression unit containing binding sites of igRNA-I_A for dSaCas9 (P_{hCMV}-a-d2EYFP-pA). oDB921 (5'-ctagcC**CAATCACAGGAGAAGGAGG***ACGAATC***CAATCACAGGAGAAGGAGG***CAGAGT*G-3') and oDB922 (5'-aatt**C***ACTCTG***CCTCCTTCTCCTGTGATTGG***ATTCGT***CCTCCTTCTCCTGTGATTGG**G-3') were annealed and inserted into *Nhe*I/*Eco*RI-digested pHK189.	This work
pDB636	Vector encoding $P_{hCMVmin}$-driven eYFP expression unit with SagRNA binding site ($P_{hCMVmin}$-a-EYFP-pA). The minimal P_{hCMV} was PCR-amplified from pDB219 with oDB966 (5'-taactcgaGCCAATCACAGGAGAAGGAGGAGGCCAATCACAGGAGAAGGAGGAGGcggccattgacaaaccgaccgtg-3') and oDB967 (5'-tatgaattCCGCGGAGGCTGGATCGGTCC-3'), digested with *Xho*I/*Eco*RI and inserted into *Xho*I/*Eco*RI-digested pDB234.	This work

Abbreviations: **Bsr,** blasticidin S-resistance gene; **BS_x,** binding site of gRNA-X; **CALR,** calreticulin; **Cas9,** CRISPR-associated protein 9; **dCas9**, catalytic inactive form of Cas9; **d2EYFP**, destabilized variant of the enhanced yellow fluorescent protein; **EGFP**, enhanced green fluorescent protein; **gRNA,** guide ribonucleic acid; **iRFP,** infrared fluorescent protein; **igRNA,** input gRNA; **KRAB**, Krueppel-associated box protein of the human *kox-1* gene; **mAID,** mini auxin-inducible degron; **mCherry,** monomeric red fluorescent protein; **MCS,** multiple cloning site; **NLS,** nuclear localization signal; **OsTIR1,** TIR1 derived from Oryza sativa; **pA,** polyadenylation signal; **PAM,** protospacer adjacent motif; **P_{hCMV},** human cytomegalovirus immediate early promoter; **P_{hU6}**, human U6 RNA polymerase III promoter; **rgRNA,** regulatory gRNA; **SaCas9,** *Staphylococcus aureus* Cas9; **SagRNA,** *Staphylococcus aureus* gRNA; **SpCas9,** *Streptococcus pyogenes* Cas9, **SpgRNA,** *Streptococcus pyogenes* gRNA**; TetO,** bacterial tet operator**; TEV,** Tobacco Etch Virus nuclear-inclusion-a endopeptidase**; tRNA,** transfer ribonucleic acid; **VPR,** potent transcriptional activator

Oligonucleotides: Restriction endonuclease-specific sites are underlined, annealing base pairs are indicated in capital letters, the gRNA binding site is shown in bold, and PAM sequence is shown in italic.

Supplementary Table 2. Composition of the transfected synthetic logic gate components in this work. Composition of the OFF-System (a), ON-System (b), A NOR B (c), A NIMPLY B (d), A AND B (e), A XOR B (f), Half adder (g), dual core ON-System (h), and dual core B NIMPLY A (i) components transfected into HEK-293T cells. To standardize transfection conditions, all component mixtures were adjusted to equal DNA concentrations by addition of an inert filler plasmid (pcDNA3.1(+) or pUC19; New England Labs Inc., United Kingdom). (see Table S1 for detailed description of vector genetics.)

a. Composition of OFF-System components for transfection (Fig. 1, Supplementary Fig. 5)

Plasmid (ng)	OFF-System	
	0	1
pHK2	450	450
pHK194	0	500
pHK182	40	50
pMM581	10	10
Filler plasmid	500	0
Total amount	1000	1000

b. Composition of ON-System components for transfection (Fig. 1, Supplementary Fig. 6)

Plasmid (ng)	ON-System	
	0	1
pHK2	450	450
pHK194	0	250
pHK225	250	250
pHK185	40	40
pMM581	10	10
Filler plasmid	250	0
Total amount	1000	1000

c. Composition of NOR gate components for transfection (Fig. 2, Supplementary Fig. 12)

Plasmid (ng)	A NOR B			
	00	10	01	11
pHK2	450	450	450	450
pHK194	0	250	0	125
pHK202	0	0	250	125
pHK216	40	40	40	40
pMM581	10	10	10	10
Filler plasmid	500	250	250	250
Total amount	1000	1000	1000	1000

d. Composition of NIMPLY gate components for transfection (Fig. 2, Supplementary Fig. 13)

Plasmid (ng)	A NIMPLY B			
	00	10	01	11
pHK2	450	450	450	450
pHK194	0	250	0	125
pHK202	0	0	250	125
pHK225	250	250	250	250
pHK221	40	40	40	40
pMM581	10	10	10	10
Filler plasmid	250	0	0	0
Total amount	1000	1000	1000	1000

e. Composition of AND gate components for transfection (Fig. 2, Supplementary Fig. 14)

Plasmid (ng)	A AND B			
	00	10	01	11
pHK2	300	300	300	300
pHK194	0	250	0	125
pHK202	0	0	250	125
pHK203	250	250	250	250
pHK225	250	250	250	250
pHK222	90	90	90	90
pMM581	10	10	10	10
Filler plasmid	250	0	0	0
Total amount	1150	1150	1150	1150

f. Composition of XOR gate components for transfection (Fig. 2, Supplementary Fig. 15)

Plasmid (ng)	A XOR B			
	00	10	01	11
pHK2	300	300	300	300
pHK194	0	250	0	125
pHK202	0	0	250	125
pHK203	250	250	250	250
pHK225	250	250	250	250
pHK212	100	100	100	100
pHK221	40	40	40	40
pMM581	10	10	10	10
Filler plasmid	250	0	0	0
Total amount	1200	1200	1200	1200

g. Composition of half adder components for transfection (Fig. 2, Supplementary Fig. 16)

Plasmid (ng)	Half adder			
	00	10	01	11
pHK2	1200	1200	1200	1200
pHK194	0	800	0	400
pHK202	0	0	800	400
pHK203	800	800	800	800
pHK225	800	800	800	800
pHK212	200	200	200	200
pHK221	80	80	80	80
pHK229	100	100	100	100
pMM581	20	20	20	20
Filler plasmid	800	0	0	0
Total amount	4000	4000	4000	4000

h. Composition of dual core ON - switch (Fig. 3a)

Plasmid (ng)	Dual core ON-Switch	
	0	1
pHK2	187.5	187.5
pHK202	0	187.5
pDB613	62.5	62.5
pHK315	62.5	62.5
pHK292	5	5
pMM581	5	5
Filler plasmid	187.5	0
Total amount	510	510

i. Composition of dual core B NIMPLY A - gate (Fig. 3b, Supplementary Fig. 18)

Plasmid (ng)	Dual core NIMPLY - gate			
	00	10	01	11
pHK2	187.5	187.5	187.5	187.5
pHK194	0	187.5	0	93.75
pHK202	0	0	187.5	93.75
pDB613	62.5	62.5	62.5	62.5
pHK315	62.5	62.5	62.5	62.5
pHK316	5	5	5	5
pMM581	5	5	5	5
Filler plasmid	187.5	0	0	0
Total amount	510	510	510	510

Supplementary Table 3. gRNA targeting sequences

gRNA index	gRNA binding site	Sequence (5'-3')
IgRNA-I_A	a	CCAATCACAGGAGAAGGAGG
IgRNA-I_B	b	GCTGACAACCTGCAGCACCA
IgRNA- I_C	c	AACCTGCTGCAGCAGCCGCG
IgRNA- I_D	d	CGTCTTCTCCAGGATCACAA
RgRNA-R_1	r_1	CGTGCGAATCTGGTGGCACC
RgRNA-R_2	r_2	TCTGTTGTGTGACTCTGGTA
RgRNA-R3	r_3	CATTGGACACAGAATCCGAG

Supplementary Table 4.

a. Composition of DNA components for the transfection to compare the suppressive effects of dCas9 and dCas9-KRAB (Supplementary Fig. 1).

Plasmid (ng)	dCas9 vs dCas9-KRAB					
dCas9-KRAB	0	25	0	0	25	0
dCas9	0	0	25	0	0	25
pHK194	0	0	0	300	300	300
pNeuLite	300	300	300	300	300	300
Filler plasmid	700	675	675	400	375	375
Total amount	1000	1000	1000	1000	1000	1000

b. Composition of DNA components for the transfection to compare the suppressive effects of dCas9-KRAB and activation effect of dCas9-VP64 (Supplementary Fig. 2).

Plasmid (ng)	Repression vs. Activation	
	0	1
pHK2 / pcDNA-dCas9-VP64	245	245
pHK194	0	245
pHK182 / pDB636	5	5
pMM581	5	5
Filler plasmid	245	0
Total amount	500	500

c. Composition of DNA components for the transfection to confirm the tRNA processed gRNA-expressing constructs (Supplementary Fig. 3).

Plasmid (ng)	IgRNA-I_A		
	mock	gRNA(I)	gRNA(II)
pHK2	450	450	450
pJP4	0	500	0
pHK194	0	0	500
pHK182	50	50	50
Filler plasmid	500	0	0
Total amount	1000	1000	1000

Plasmid (ng)	IgRNA-I_B		
	mock	gRNA(I)	gRNA(II)
pHK2	450	450	450
pHK56	0	500	0
pHK202	0	0	500
pHK62	50	50	50
Filler plasmid	500	0	0
Total amount	1000	1000	1000

Plasmid (ng)	$RgRNA-R_1$		
	mock	gRNA(I)	gRNA(II)
pHK2	450	450	450
pJP47	0	500	0
pHK197	0	0	500
pHK185	50	50	50
Filler plasmid	500	0	0
Total amount	1000	1000	1000

Plasmid (ng)	$RgRNA-R_2$		
	mock	gRNA(I)	gRNA(II)
pHK2	450	450	450
pJP52	0	500	0
pHK201	0	0	500
pHK189	50	50	50
Filler plasmid	500	0	0
Total amount	1000	1000	1000

d. Composition of DNA components for the transfection to confirm the enhanced activity of tRNA processed regulatory rgRNA-expressing constructs (Supplementary Fig. 4).

Plasmid (ng)	No tRNA vs. tRNA						
	Mock	pHK75	pHK141	pHK76	pHK142	pHK77	pHK143
pHK2	150	150	150	150	150	150	150
pHK63	5	5	5	5	5	5	5
Variable	0	175	175	175	175	175	175
pMM581	5	5	5	5	5	5	5
pU6	350	175	175	175	175	175	175
total amount	510	510	510	510	510	510	510

e. Composition of DNA components for the transfection to confirm the time course (Supplementary Fig. 7).

Plasmid (ng)	OFF-System	
	0	1
pHK2	225	225
pHK194	0	250
pHK182	20	20
pMM581	5	5
Filler plasmid	250	0
Total amount	500	500

f. Composition of DNA components for the transfection to confirm dose-dependent activity (Supplementary Fig. 8).

Plasmid (ng)	Dose-Dependence OFF					
pHK2	225	225	225	225	225	225
pHK194	0	50	100	150	200	250
pHK182	20	20	20	20	20	20
pMM581	5	5	5	5	5	5
Filler plasmid	250	200	150	100	50	0
Total amount	500	500	500	500	500	500

g. Composition of DNA components for the transfection to confirm degron activity (Supplementary Fig. 9).

Plasmid (ng)	Degron			
	OFF-0	OFF-1	ON-0	ON-1
pHK2	225	225	225	225
pHK310	20	20	0	0
pHK311	0	0	20	20
pHK194	0	250	0	125
pHK225	0	0	125	125
pMM581	5	5	5	5
Filler plasmid	250	0	125	0
Total amount	500	500	500	500

h. Composition of DNA components for the time-delay transfection to test the inhibitory effect of rgRNA-R_1 by igRNA-I_A (Supplementary Fig. 10).

Plasmid (ng)	Co-transfection		Time-delay transfection	
	0	1	0	1
pHK2	450	450	450	450
pHK194	0	250	0	250
pHK225	250	250	250	250
pHK185	40	40	40	40
pMM581	10	10	10	10
Filler plasmid	250	0	250	0
total amount	1000	1000	1000	1000

i. Composition of DNA components for transfection to test orthogonal repression activity (Supplementary Fig. 11)

Plasmid (ng)	IgRNA-I_A (a)				
	Mock	igRNA-I_A	igRNA-I_B	rgRNA-R_1	rgRNA-R_2
pHK2	300	300	300	300	300
pJP4	0	300	0	0	0
pHK56	0	0	300	0	0
pJP47	0	0	0	300	0
pJP52	0	0	0	0	300
pHK182	30	30	30	30	30
Filler plasmid	300	0	0	0	0
total amount	630	630	630	630	630

Plasmid (ng)	IgRNA-I$_B$ (b)				
	Mock	igRNA-I$_A$	igRNA-I$_B$	rgRNA-R$_1$	rgRNA-R$_2$
pHK2	300	300	300	300	300
pJP4	0	300	0	0	0
pHK56	0	0	300	0	0
pJP47	0	0	0	300	0
pJP52	0	0	0	0	300
pHK62	30	30	30	30	30
Filler plasmid	300	0	0	0	0
total amount	630	630	630	630	630

Plasmid (ng)	RgRNA-R$_1$ (c)				
	Mock	igRNA-I$_A$	igRNA-I$_B$	rgRNA-R$_1$	rgRNA-R$_2$
pHK2	300	300	300	300	300
pJP4	0	300	0	0	0
pHK56	0	0	300	0	0
pJP47	0	0	0	300	0
pJP52	0	0	0	0	300
pHK185	30	30	30	30	30
Filler plasmid	300	0	0	0	0
total amount	630	630	630	630	630

Plasmid (ng)	RgRNA-R$_2$ (d)				
	Mock	igRNA-I$_A$	igRNA-I$_B$	rgRNA-R$_1$	rgRNA-R$_2$
pHK2	300	300	300	300	300
pJP4	0	300	0	0	0
pHK56	0	0	300	0	0
pJP47	0	0	0	300	0
pJP52	0	0	0	0	300
pHK189	30	30	30	30	30
Filler plasmid	300	0	0	0	0
total amount	630	630	630	630	630

j. Composition of DNA components for transfection to test orthogonal repression by dSaCas9-KRAB (Supplementary Fig. 17)

Plasmid (ng)	Dose-Dependence DSaCas9-KRAB					
pDB613	225	225	225	225	225	225
pDB615	0	50	100	150	200	250
pDB616	20	20	20	20	20	20
pMM581	5	5	5	5	5	5
Filler plasmid	250	200	150	100	50	0
Total amount	500	500	500	500	500	500

Supplementary Figures

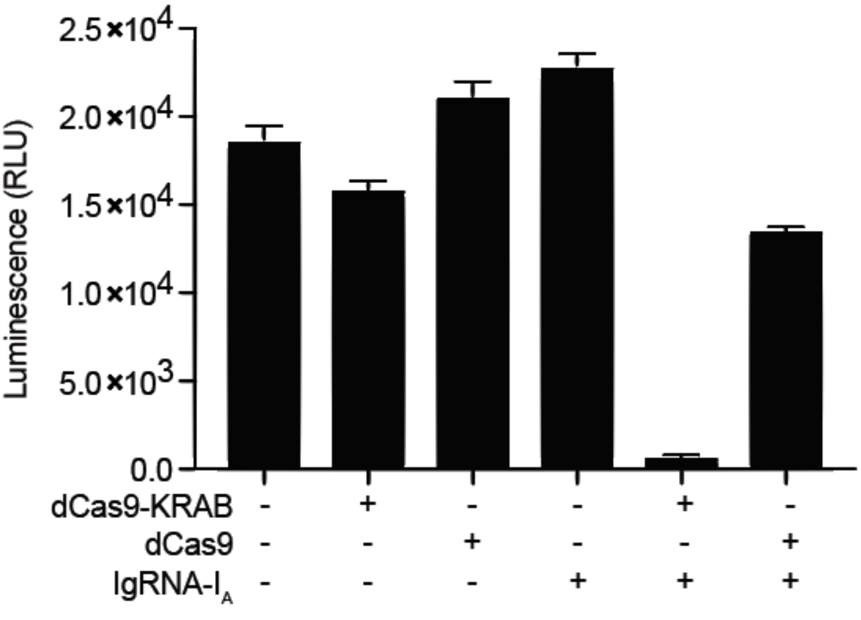

Supplementary Figure 1. Comparison of the suppressive effects of dSpCas9 and dSpCas9-KRAB. Transient transfection of SpgRNAs repressed reporter gene expression in HEK-293T cells. Cells were transfected with the indicated plasmids (Supplementary Table 4) and analyzed by Envision reader for luciferase expression 48 hours post-transfection. The data are displayed as means ± s.d. for three independent transfections (*n* = 3). Mean luminescence intensities are presented as arbitrary units (RLU).

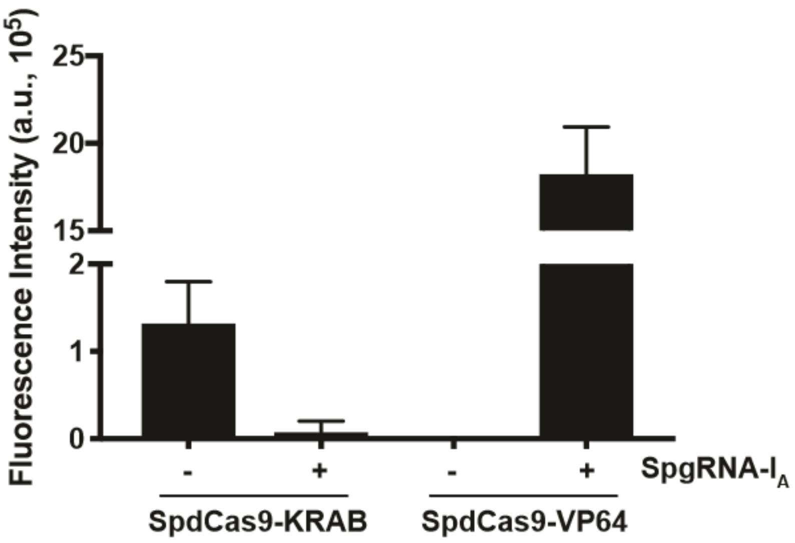

Supplementary Figure 2. Comparison of the repression-based (dSpCas9-KRAB) and the activation-based (dSpCas9-VP64) OFF-switch. Transient transfection of gRNAs repressed or activated reporter gene expression in HEK-293T cells. Cells were transfected with the indicated plasmids (Supplementary Table 4) and analyzed by FACS for d2EYFP expression 48 hours post-transfection. The data are displayed as means ± s.d. for three independent transfections ($n = 3$). Mean fluorescence intensities are presented as arbitrary units (a.u.).

a

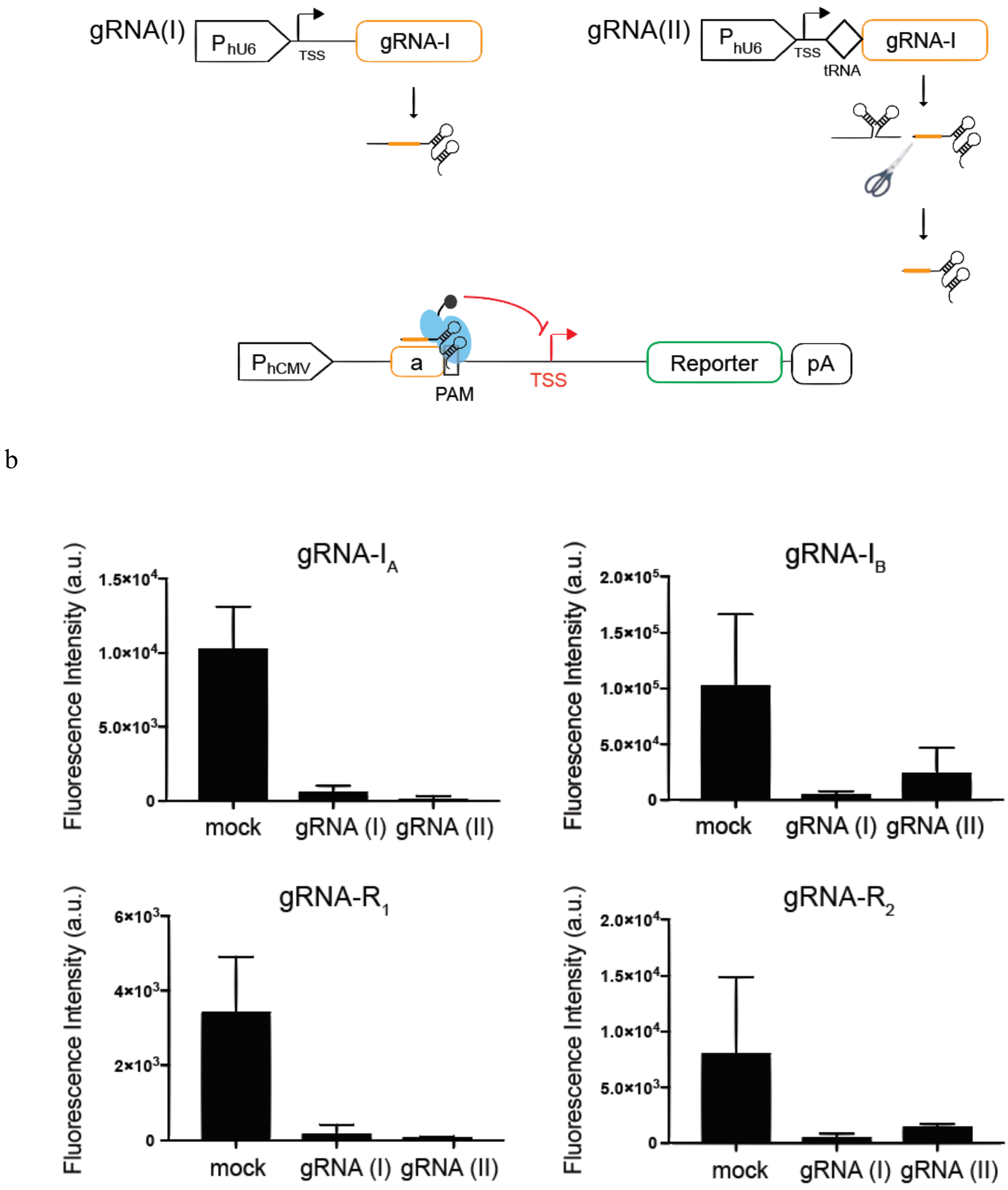

Supplementary Figure 3. Influence of tRNA processing gRNA-expressing constructs. a, Diagram of the gRNA-expressing construct without tRNA (I) or with tRNA (II) and a reporter gene construct that contains gRNA-binding site to regulate d2EYFP transcription. tRNA can be processed by intrinsic cellular proteins RNase P and RNase Z to produce gRNA carrying the desired 5′-target sequences without extra nucleotides. **b,** Fluorescent protein quantification 48 h post-transfection for HEK-293T cells transfected with expression plasmids for dSpCas9-KRAB, the reporter gene with corresponding binding sites for SpgRNA and the SpgRNA with or without tRNA architecture (Supplementary Table 4). tRNA-conjugated SpgRNA showed comparable repression activity to the SpgRNAs without tRNA for four different specific targets. The data are displayed as means ± s.d. for three independent transfections (n = 3). Mean fluorescence intensities are presented as arbitrary units (a.u.).

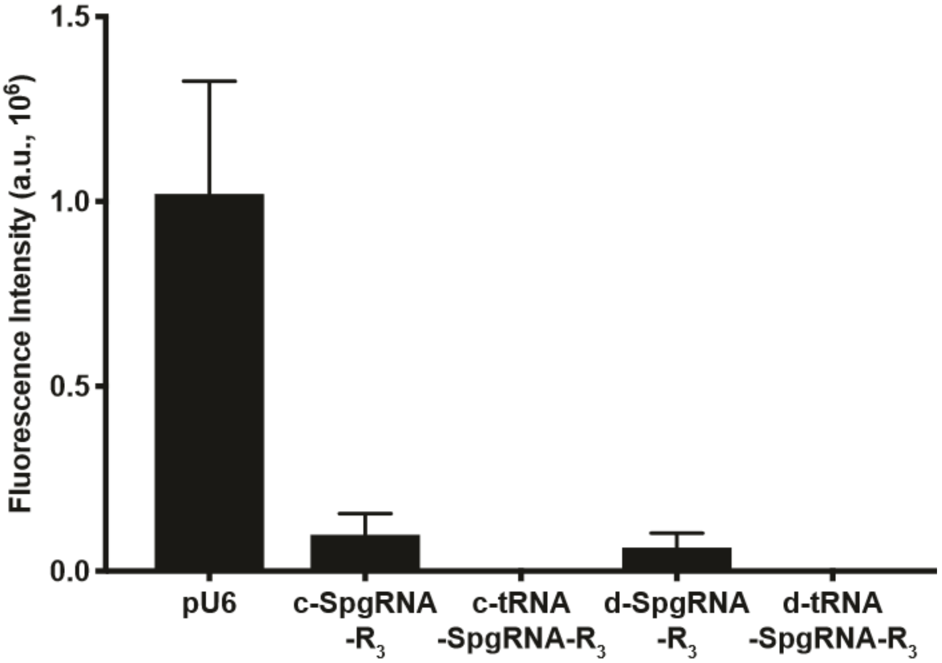

Supplementary Figure 4. Influence of tRNA processing on regulatory gRNA activity. Fluorescent protein quantification 48 h post-transfection for HEK-293T cells transfected with expression plasmids for dSpCas9-KRAB, the reporter gene with corresponding binding sites for SpgRNA and the regulatory SpgRNAs with or without tRNA architecture (Supplementary Table 4). tRNA-conjugated regulatory SpgRNAs (pHK141 and pHK142) showed higher repression activity in comparison to the SpgRNAs without tRNA (pHK75 and pHK76) for two different constructs. The data are displayed as means ± s.d. for three independent transfections (*n* = 3). Mean fluorescence intensities are presented as arbitrary units (a.u.).

a

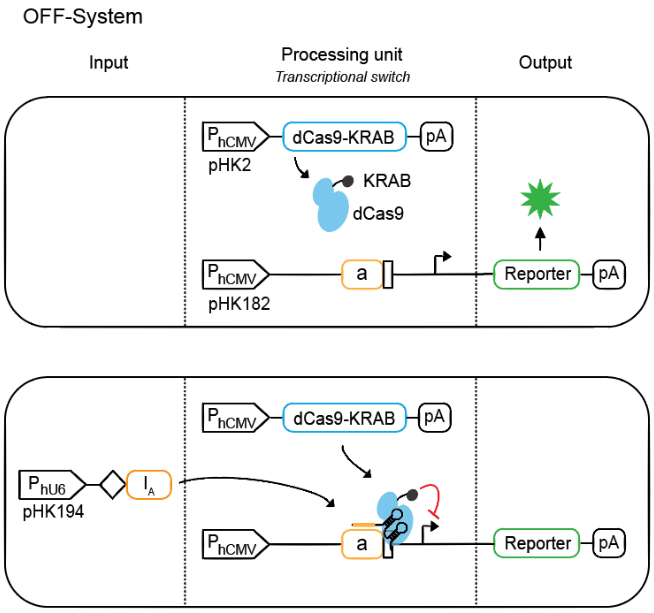

b

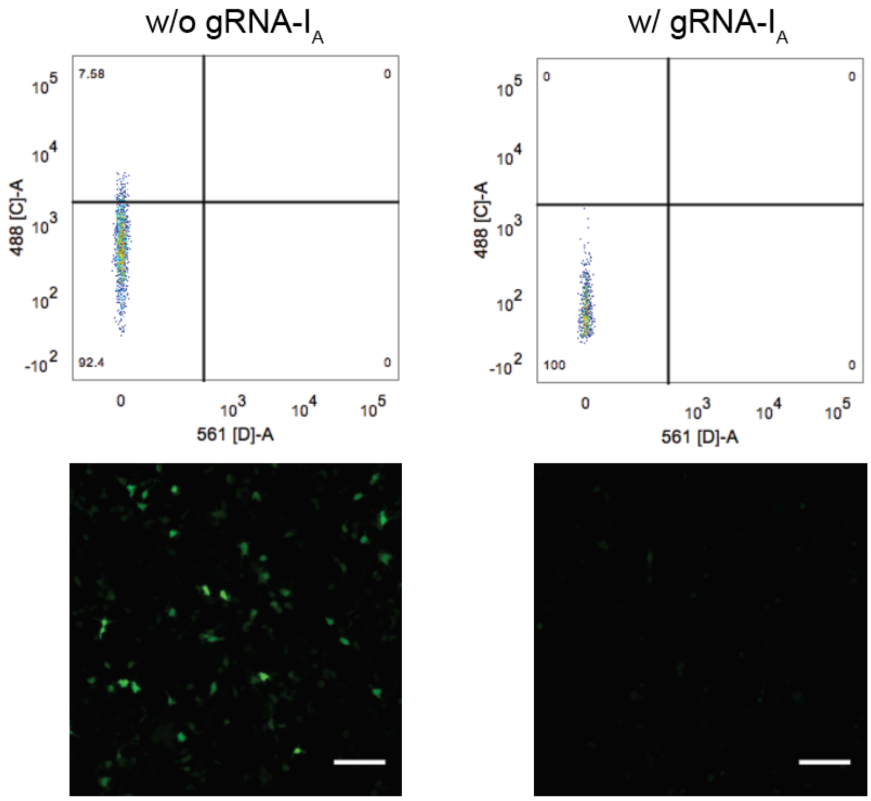

Supplementary Figure 5. Raw flow cytometry data of the OFF-system. a, Schematic representation of genetic circuit components and dynamics of the OFF-system in relation to the presence of igRNA-I$_A$. **b,** Raw flow cytometry data and fluorescent images (excitation wavelength: 513/17 nm; scale bar = 100 µm) of the OFF-system after gating. Fluorescence of d2EYFP driven by P$_{hCMV}$ promoter with igRNA-I$_A$ binding site (pHK182) inside gate P3, shown for d2EYFP (488 nm) and mCherry (561 nm) filter sets and within the respective quadrants. The d2EYFP-positive cell population Q1 is presented as the output.

a

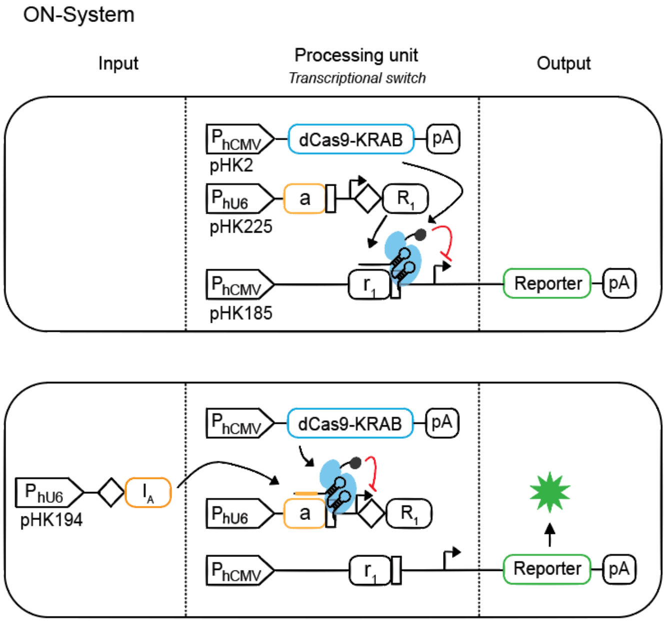

b

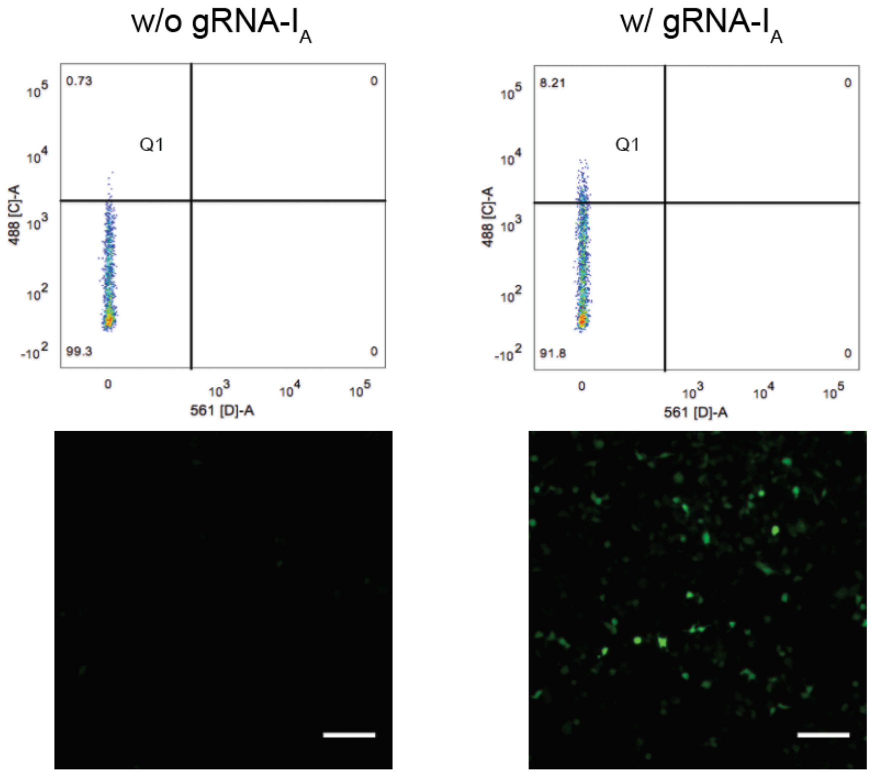

Supplementary Figure 6. Raw flow cytometry data of the ON-system. a, Schematic representation of gene circuit components and dynamics of the ON-system in relation to the presence of igRNA-I_A. **b,** Raw flow cytometry data and fluorescent images (excitation wavelength: 513/17 nm; scale bar = 100 µm) of the ON-system after gating. Fluorescence of d2EYFP driven by a P_{hCMV} promoter with rgRNA-R_1 binding site (pHK185) inside gate P3, shown for d2EYFP (488 nm) and mCherry (561

nm) filter sets and within the respective quadrants. The d2EYFP-positive cell population Q1 is presented as the output.

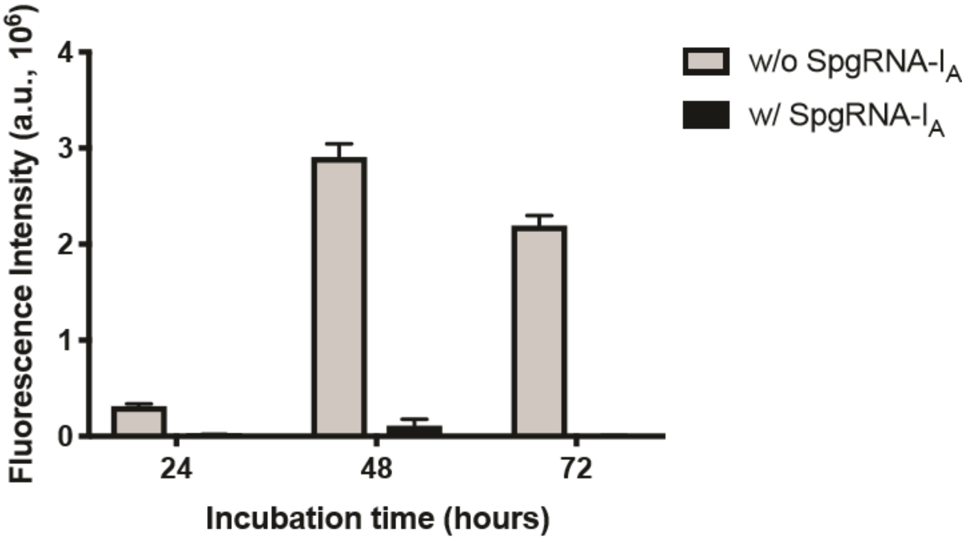

Supplementary Figure 7. Time course measurement of the OFF-switch. Fluorescent protein quantification 24 h, 48 h and 72 h post-transfection for HEK-293T cells transfected with expression plasmids for igRNA-I_A, dSpCas9-KRAB and the reporter gene with corresponding binding sites for igRNA. The data are displayed as means ± s.d. for three independent transfections (*n* = 3). Mean fluorescence intensities are presented as arbitrary units (a.u.).

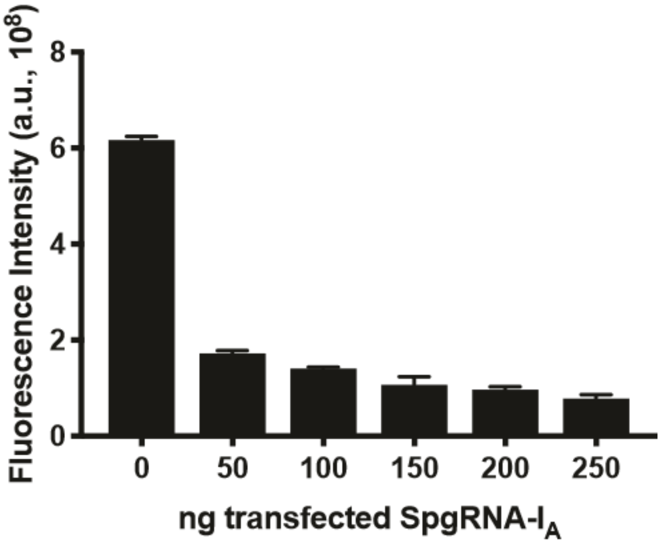

Supplementary Figure 8. Dose-dependent performance of the OFF-switch.. Fluorescent protein quantification 48 h post-transfection for HEK-293T cells transfected with expression plasmids for different doses of igRNA-I_A, dSpCas9-KRAB and the reporter gene with corresponding binding sites for igRNA-I_A. The data are displayed as means ± s.d. for three independent transfections (*n* = 3). Mean fluorescence intensities are presented as arbitrary units (a.u.).

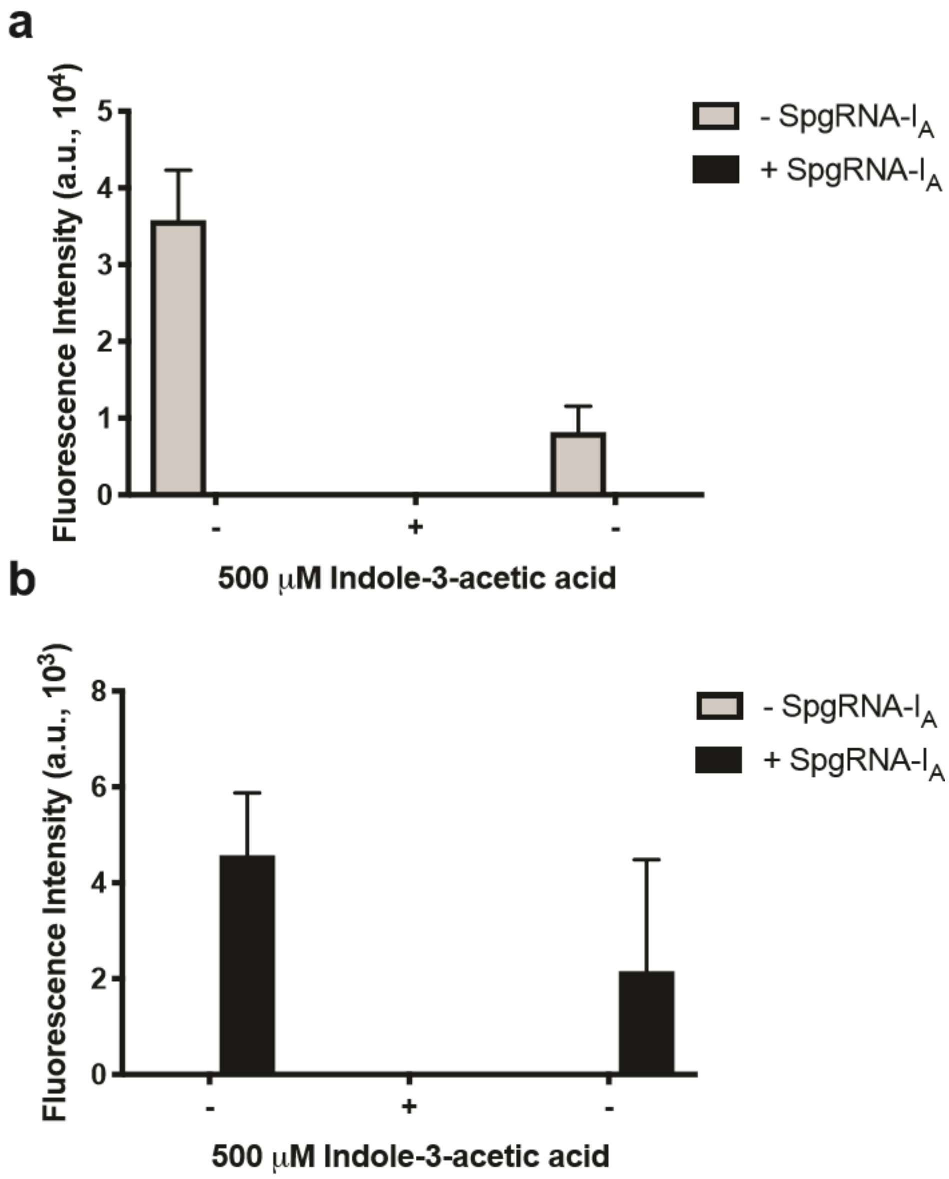

Supplementary Figure 9. Auxin-induced degron for switching off the system. Fluorescent protein was quantified in one set of wells (left column) at 48 h post-transfection for HEK-293T cells transfected with the indicated plasmids (Supplementary Table 4) for the degron-modified OFF-switch (**a**) or ON-switch (**b**). Then 500 µM indole-3-acetic acid (IAA, in ethanol, SigmaAldrich I3750-5G-A) were added to the remaining wells. Four hours later, fluorescence was measured in the next set of wells (middle column). The last set of wells was washed twice with normal DMEM, the medium was exchanged to DMEM, and six hours later, the fluorescence in these wells was measured (right column). The data are displayed as means ± s.d. for three independent transfections (*n* = 3). Mean fluorescence intensities are presented as arbitrary units (a.u.).

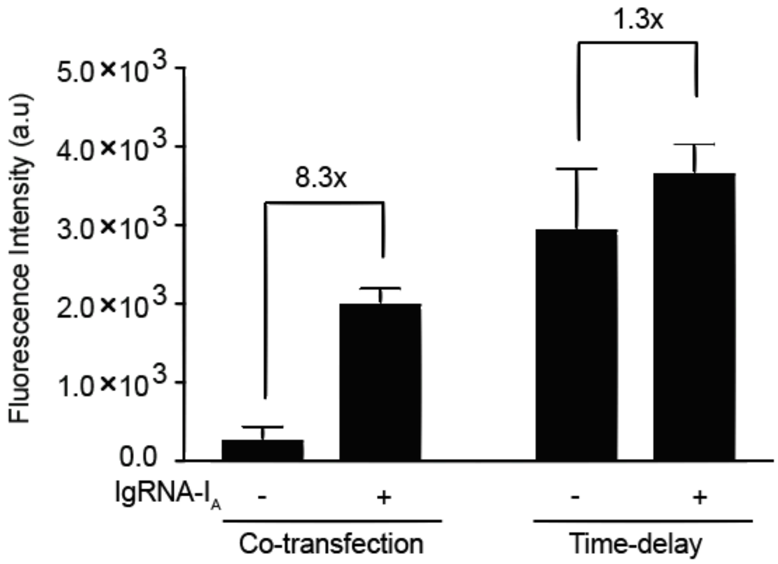

Supplementary Figure 10. Inhibitory effect of rgRNA-R_1 by igRNA-I_A. d2EYFP expression was re-activated when rgRNA-R_1 was repressed by iRNA-I_A. We co-transfected igRNA-I_A (pHK194) and rgRNA-R_1 (pHK225) or introduced them by a sequential transfection with 6 hour time-delay. Fluorescent protein quantification was performed at 48 h post-transfection for HEK-293T cells transfected with the indicated plasmids (Supplementary Table 4). The data are displayed as means ± s.d. for three independent transfections (n = 3). Mean fluorescence intensities are presented as arbitrary units (a.u.).

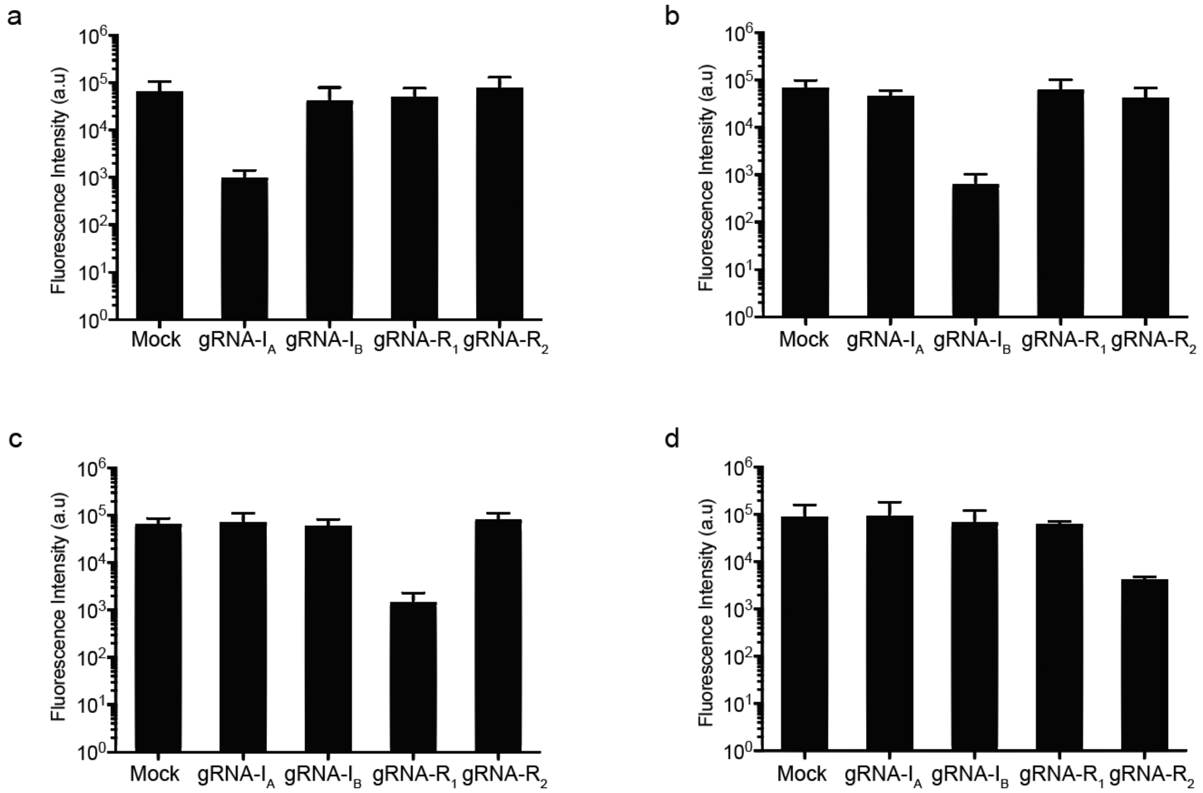

Supplementary Figure 11. Orthogonal repression activity of input and regulatory gRNAs. Four guide RNAs repressed d2EYFP expression when matched to their corresponding binding sites on the promoter of reporter plasmids; IgRNA-I_A (a, pHK182), IgRNA-I_B (b, pHK62), RgRNA-R_1 (c, pHK185) or RgRNA-R_2 (d, pHK189). Fluorescent protein quantification at 48 h post-transfection for HEK-293T cells transfected with the indicated plasmids (Supplementary Table 4), the expression plasmids for the reporter gene with corresponding binding sites for gRNA and the gRNAs in the orthogonality matrix. The data are displayed as means ± s.d. for three independent transfections (*n* = 3). Mean fluorescence intensities are presented as arbitrary units (a.u.).

a

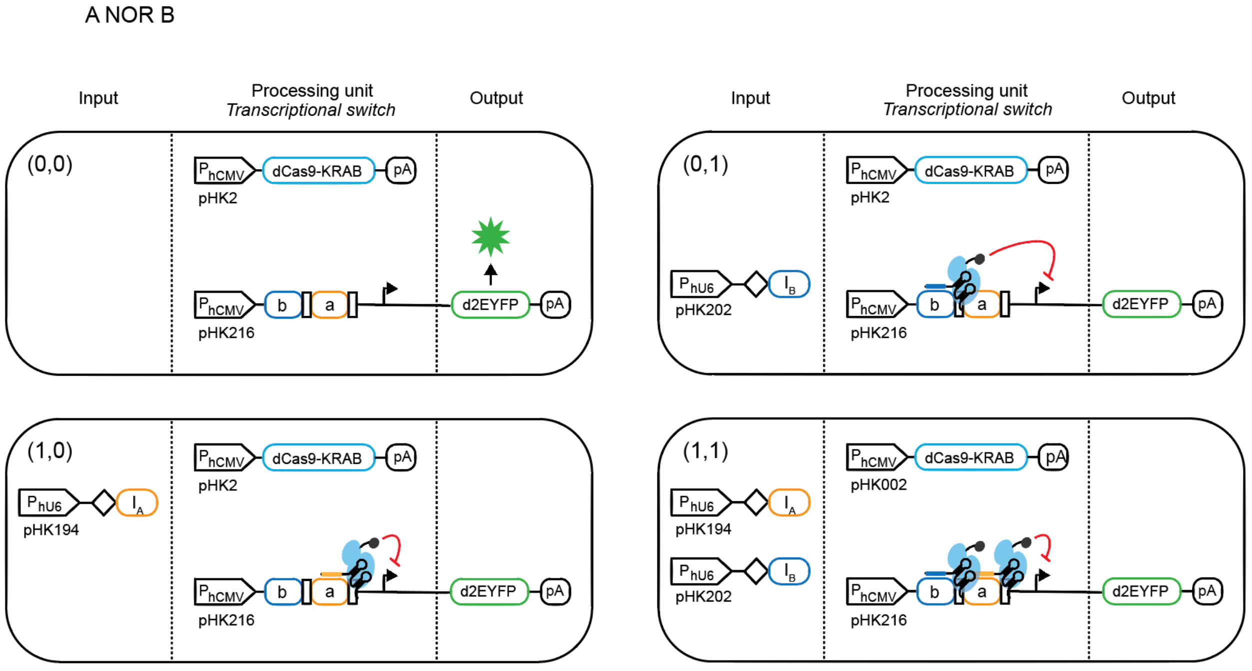

b

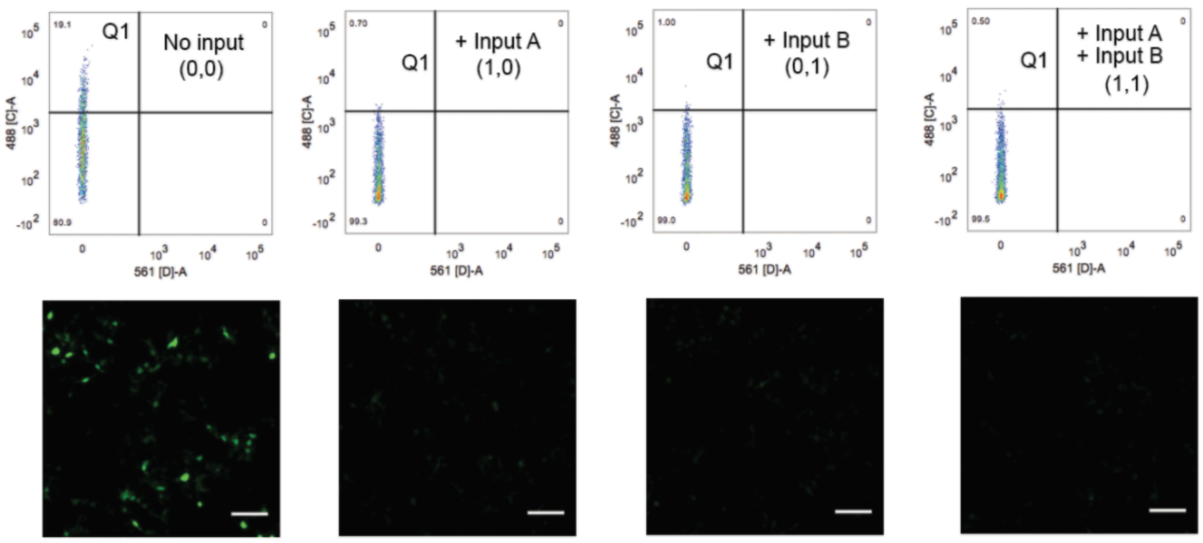

Supplementary Figure 12. Schematic of the NOR gate processing unit and raw flow cytometry data. a, Schematic representation of gene circuit components and dynamics of the A NOR B gate in relation to the presence of IgRNAs. **b,** Raw flow cytometry analysis data and fluorescent images (excitation wavelength: 513/17 nm; scale bar = 100 μm) of the performance of the A NOR B gate. Fluorescence of d2EYFP driven by a P_{hCMV} promoter with IgRNA-I_A and -I_B binding sites (pHK216) inside gate P3, shown for d2EYFP (488 nm) and mCherry (561 nm) filter sets and within the respective quadrants. The d2EYFP-positive cell population Q1 is presented as the output.

a

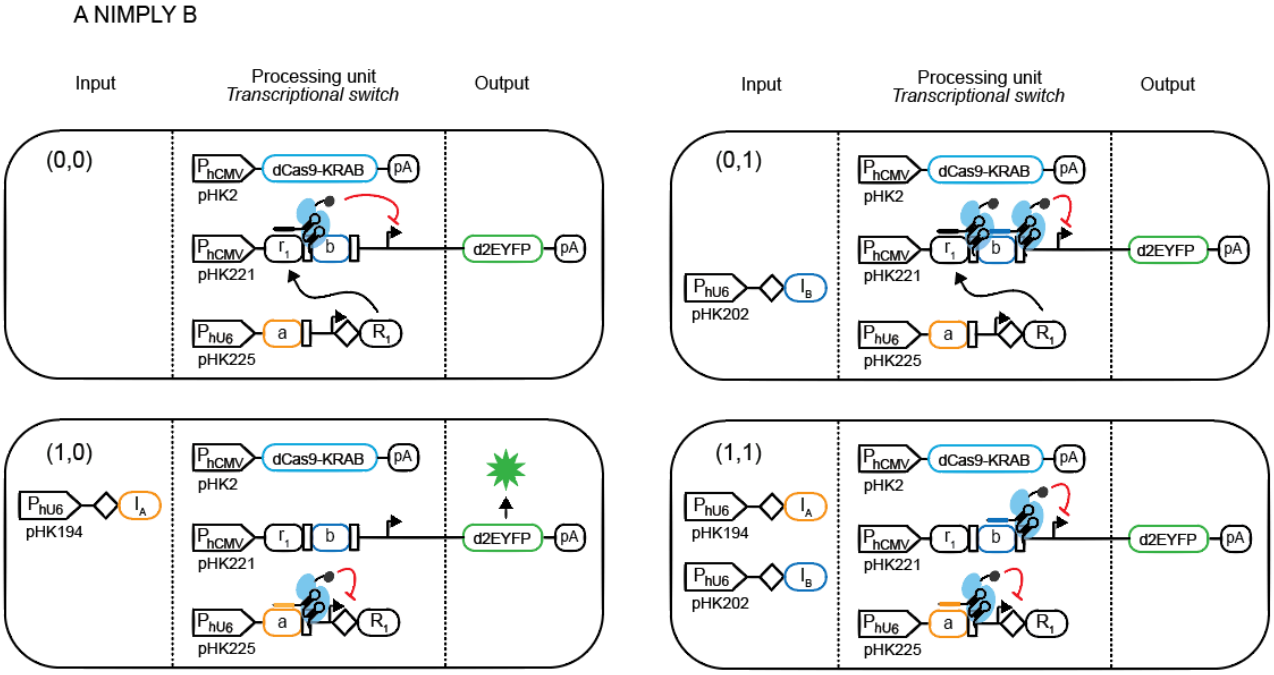

b

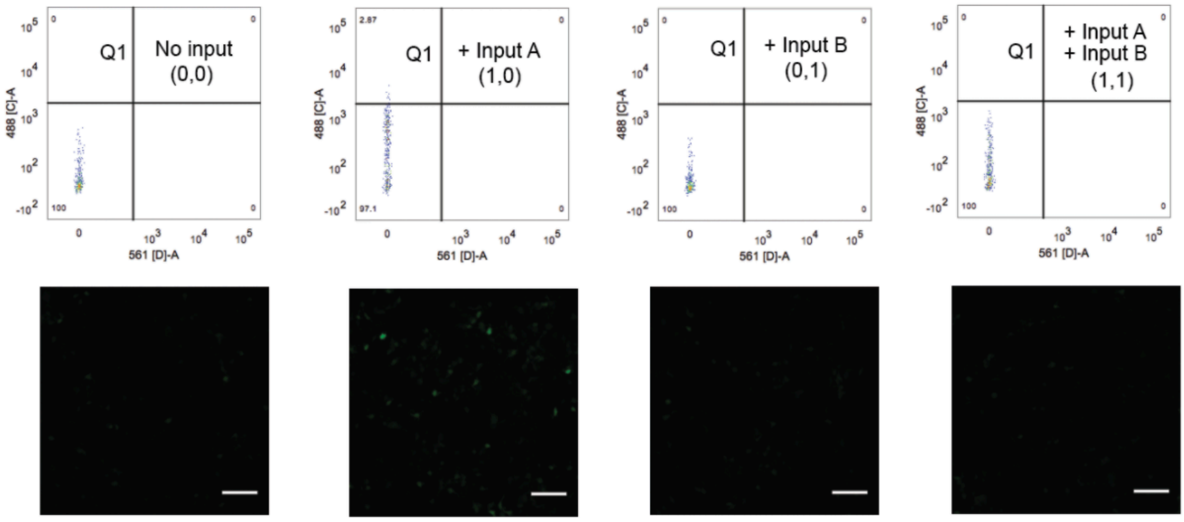

Supplementary Figure 13. Schematic of the NIMPLY gate processing unit and raw flow cytometry data. a, Schematic representation of gene circuit components and dynamics of the A NIMPLY B gate in relation to the presence of igRNAs. **b,** Raw flow cytometry analysis data and fluorescent images (excitation wavelength: 513/17 nm; scale bar = 100 µm) of the performance of the A NIMPLY B gate. Fluorescence of d2EYFP driven by a P_{hCMV} promoter with IgRNA-I_B and RgRNA-R_1 binding sites (pHK221) inside gate P3, shown for d2EYFP (488 nm) and mCherry (561 nm) filter sets and within the respective quadrants. The d2EYFP-positive cell population Q1 is presented as the output.

a

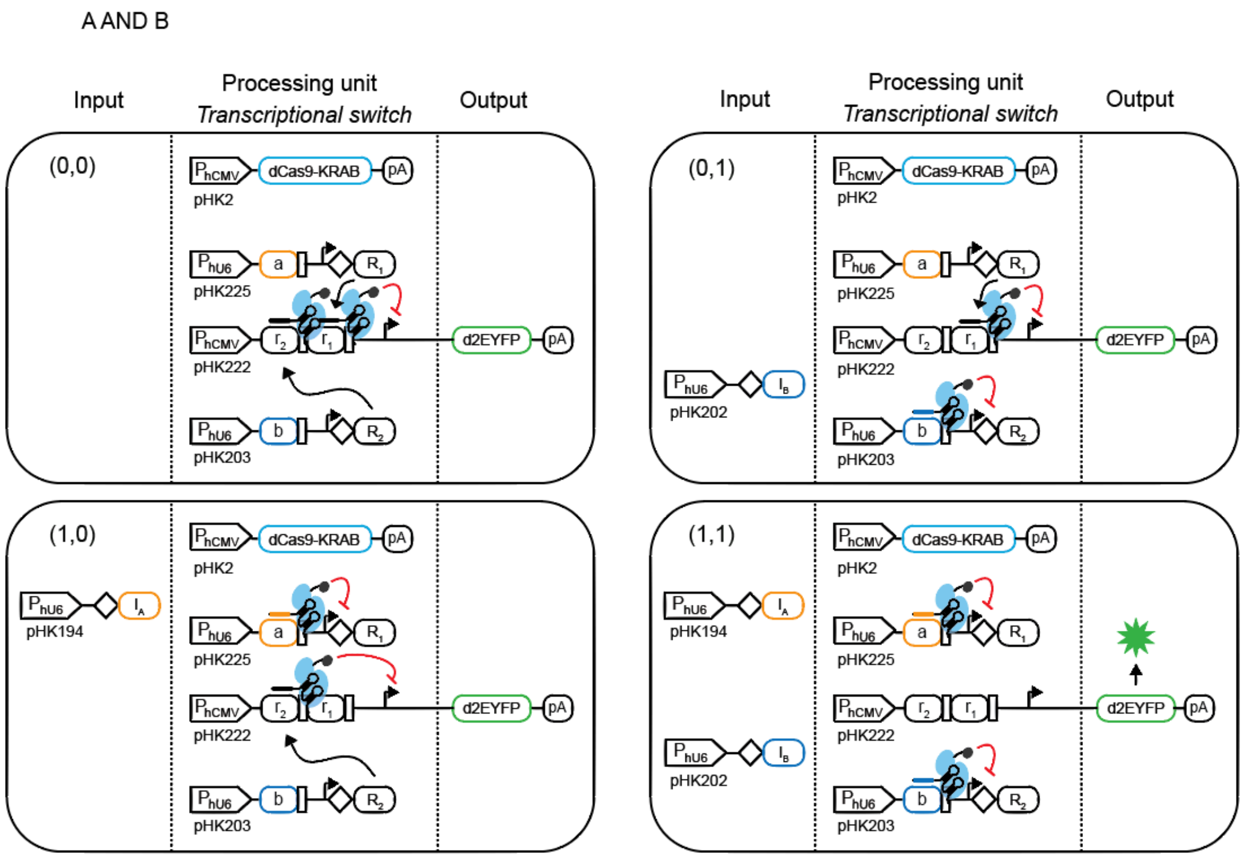

b

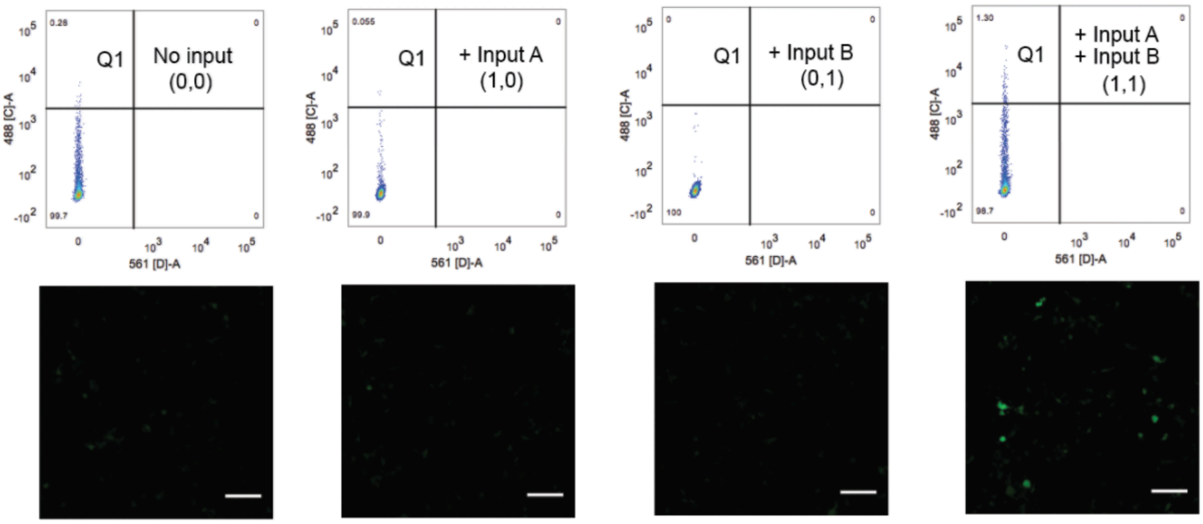

Supplementary Figure 14. Schematic of the AND gate processing unit and raw flow cytometry data. a, Schematic representation of gene circuit components and dynamics of the A AND B gate in relation to the presence of IgRNAs. **b,** Raw flow cytometry analysis data and fluorescent images (excitation wavelength: 513/17 nm; scale bar = 100 µm) of the performance of the A AND B gate. Fluorescence of d2EYFP driven by a P_{hCMV} promoter with RgRNA-R_1 and -R_2 binding sites (pHK222)

inside gate P3, shown for d2EYFP (488 nm) and mCherry (561 nm) filter sets and within the respective quadrants. The d2EYFP-positive cell population Q1 is presented as the output.

a

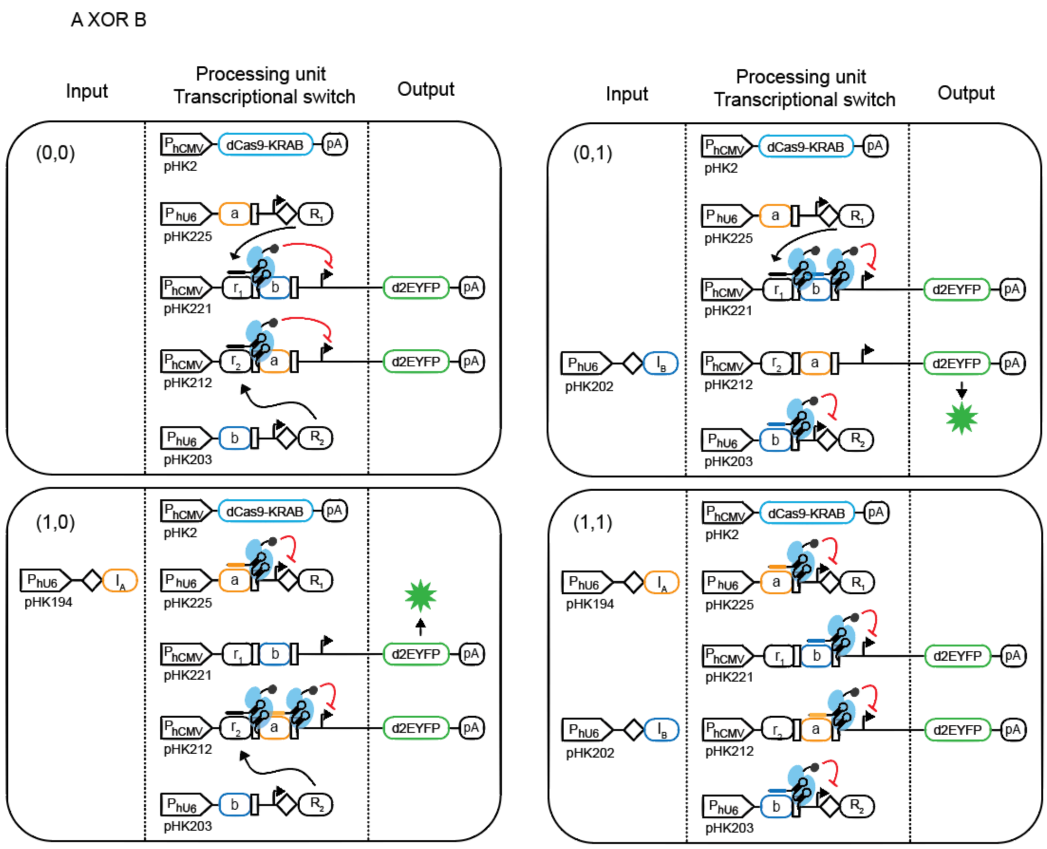

b

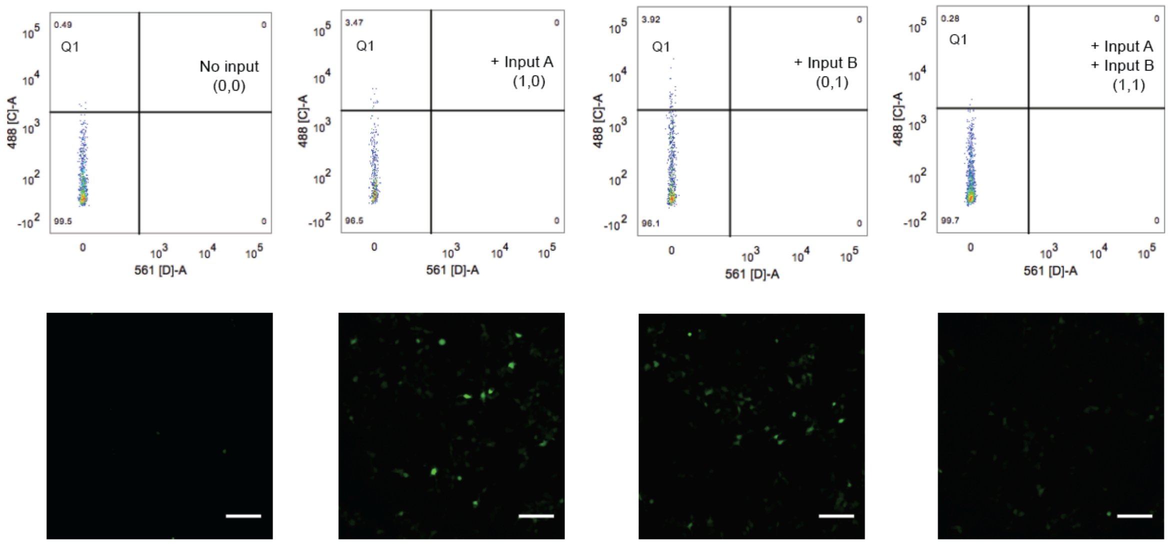

Supplementary Figure 15. Schematic of the XOR gate processing unit and raw flow cytometry data. Schematic representation of gene circuit components and dynamics of the A XOR B gate in relation to the presence of IgRNAs. **b,** Raw flow cytometry analysis data and fluorescent images (excitation wavelength: 513/17 nm; scale bar = 100 μm) of the performance of the A XOR B gate. Fluorescence of d2EYFP driven by a P_{hCMV} promoter with IgRNA-I_A and RgRNA-R_2, IgRNA-I_B and

RgRNA-R$_1$ binding sites (pHK212, pHK221) inside gate P3, shown for d2EYFP (488 nm) and mCherry (561 nm) filter sets and within the respective quadrants. The d2EYFP-positive cell population Q1 is presented as the output.

a

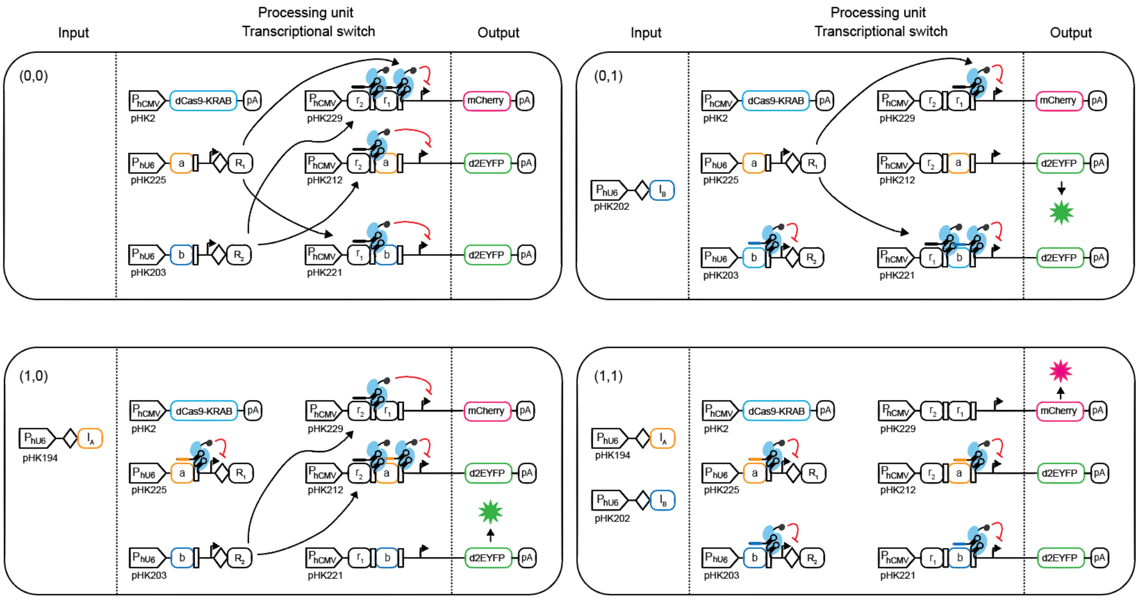

b

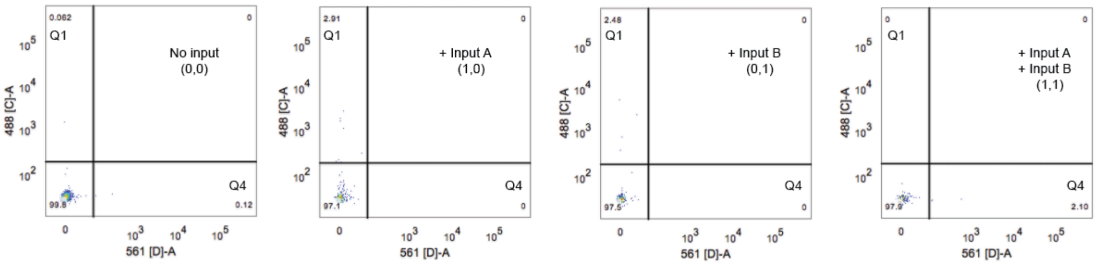

Supplementary Figure 16. Schematic of the half adder processing unit and raw flow cytometry data. a, Schematic representation of gene circuit components and dynamics of the half adder in relation to the presence of IgRNAs. **b,** Raw flow cytometry analysis data and fluorescent images (excitation wavelength: 513/17 nm and 549/15; scale bar = 100 µm) of the performance of the half adder. Fluorescence of d2EYFP (pHK212, pHK221) and mCherry (pHK229) driven by a P_{hCMV} promoter with gRNA binding sites and inside gate P3, shown for d2EYFP (488 nm) and mCherry (561 nm) filter sets and within the respective quadrants. The d2EYFP-positive cell population Q1 is presented as the sum, S_{HA}, and the mCherry-positive cell population Q4 is presented as the carry, C_{OUT}.

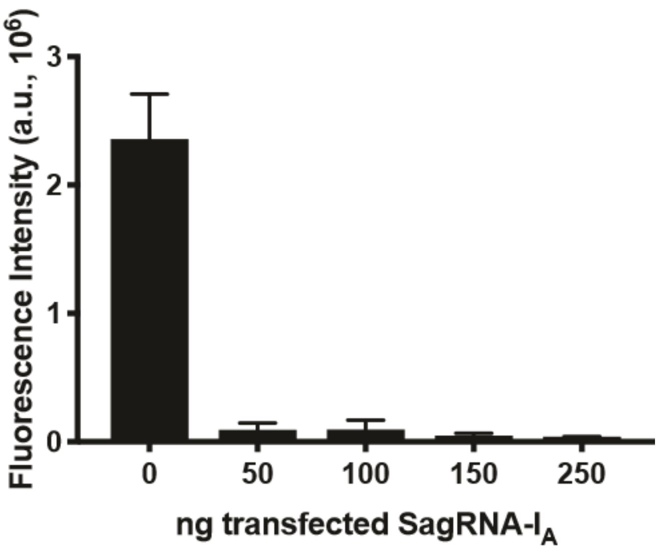

Supplementary Figure 17. Repressing gene expression with orthogonal dSaCas9-KRAB. HEK-293T cells were transfected with the indicated plasmids (Supplementary Table 4) and analyzed by FACS for d2EYFP expression 48 hours post-transfection. The data are displayed as means ± s.d. for three independent transfections (n = 3). Mean fluorescence intensities are presented as arbitrary units (a.u.).

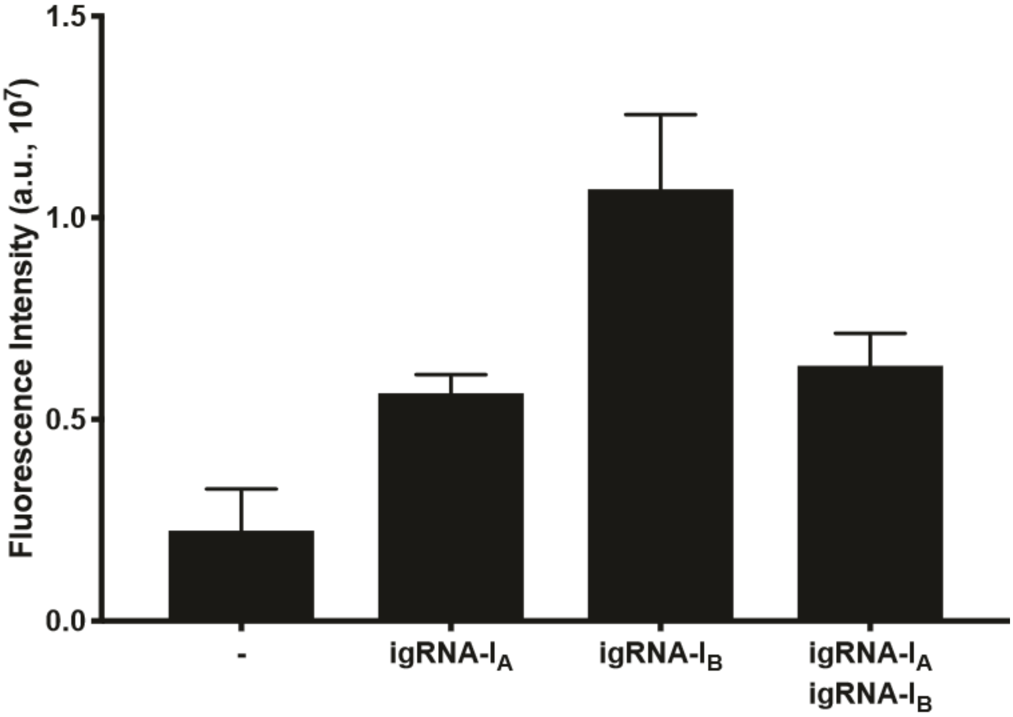

Supplementary Figure 18. Dual-core B-NIMPLY-A gate with dSpCas9-KRAB and dSaCas9-KRAB in immortalized hMSCs. Using dSpCas9-KRAB and dSaCas9-KRAB as the two processing units, the binding site for igRNA-I_B (dSpCas9-KRAB) was placed between the P_{hU6} promoter and igRNA rgRNA-R_2 and binding sites for igRNA-I_A and rgRNA-R_2 (dSaCas9-KRAB) were placed between the P_{hCMV} promoter and the reporter gene. hMSC-hTERT cells were transfected with the indicated plasmids (Supplementary Table 4) and analyzed by FACS for d2EYFP expression 48 hours post-transfection. The data are displayed as means ± s.d. for three independent transfections (*n* = 3). Mean fluorescence intensities are presented as arbitrary units (a.u.).

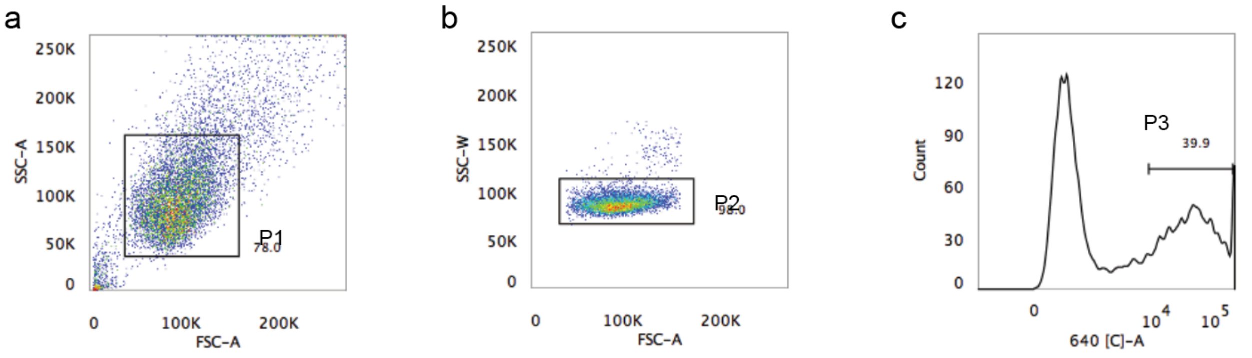

Supplementary Figure 19. Flow cytometry gating. a, Representative gating for viable HEK-293T cells via forward-scatter area (FSC-A) and side-scatter area (SSC-A), resulting in population P1. **b,** P1 population cells were gated via FSC-A and SSC-W to exclude doublet cells, resulting in population P2. **c,** Transfected cell population was gated via iRFP (640 nm) filter sets inside gate P2. Cells were analysed by flow cytometry at 48 h after transfection.

Supplementary References

1. Müller, M. et al., Designed cell consortia as fragrance-programmable analog-to-digital converters. *Nat Chem Biol* (2017).

2. Wang, H. et al., Cosmetics-triggered percutaneous remote control of transgene expression in mice. *Nucleic Acids Res* (2015).
